# Supramolecular Polymer‐Nanomedicine Hydrogel Loaded with Tumor Associated Macrophage‐Reprogramming polyTLR7/8a Nanoregulator for Enhanced Anti‐Angiogenesis Therapy of Orthotopic Hepatocellular Carcinoma

**DOI:** 10.1002/advs.202300637

**Published:** 2023-05-25

**Authors:** Xiang Liu, Yini Huangfu, Jingrong Wang, Pengxu Kong, Weijun Tian, Peng Liu, Chuang Fang, Shuangyang Li, Yu Nie, Zujian Feng, Pingsheng Huang, Shengbin Shi, Chuangnian Zhang, Anjie Dong, Weiwei Wang

**Affiliations:** ^1^ Department of Polymer Science and Engineering Key Laboratory of Systems Bioengineering (Ministry of Education) School of Chemical Engineering and Technology Tianjin University Tianjin 300072 P. R. China; ^2^ Tianjin Key Laboratory of Biomaterial Research Institute of Biomedical Engineering Chinese Academy of Medical Sciences and Peking Union Medical College Tianjin 300192 P. R. China; ^3^ Department of Structural Heart Disease Fuwai Hospital Chinese Academy of Medical Sciences and Peking Union Medical College Beijing 100037 P. R. China; ^4^ Department of General Surgery Tianjin Medical University General Hospital Tianjin 300052 P. R. China; ^5^ Department of Gastrointestinal Oncology Shandong Cancer Hospital and Institute Shandong First Medical University and Shandong Academy of Medical Sciences Jinan Shandong 250117 P. R. China; ^6^ Frontiers Science Center for Synthetic Biology Key Laboratory of Systems Bioengineering (MOE) Tianjin University Tianjin 300072 P. R. China

**Keywords:** anti‐angiogenic therapy, nanoregulator, orthotopic hepatocellular carcinoma, supramolecular hydrogel, tumor microenvironment

## Abstract

Anti‐angiogenic therapies targeting inhibition of vascular endothelial growth factor (VEGF) pathway show clinical benefit in hypervascular hepatocellular carcinoma (HCC) tumors. However, HCC expresses massive pro‐angiogenic factors in the tumor microenvironment (TME) in response to anti‐angiogenic therapy, recruiting tumor‐associated macrophages (TAMs), leading to revascularization and tumor progression. To regulate cell types in TME and promote the therapeutic efficiency of anti‐angiogenic therapy, a supramolecular hydrogel drug delivery system (PLDX‐PMI) co‐assembled by anti‐angiogenic nanomedicines (PCN‐Len nanoparticles (NPs)) and oxidized dextran (DX), and loaded with TAMs‐reprogramming polyTLR7/8a nanoregulators (p(Man‐IMDQ) NRs) is developed for orthotopic liver cancer therapy. PCN‐Len NPs target tyrosine kinases of vascular endothelial cells and blocked VEGFR signaling pathway. p(Man‐IMDQ) NRs repolarize pro‐angiogenic M2‐type TAMs into anti‐angiogenic M1‐type TAMs via mannose‐binding receptors, reducing the secretion of VEGF, which further compromised the migration and proliferation of vascular endothelial cells. On highly malignant orthotopic liver cancer Hepa1‐6 model, it is found that a single administration of the hydrogel formulation significantly decreases tumor microvessel density, promotes tumor vascular network maturation, and reduces M2‐subtype TAMs, thereby effectively inhibiting tumor progression. Collectively, findings in this work highlight the great significance of TAMs reprogramming in enhancing anti‐angiogenesis treatment for orthotopic HCC, and provides an advanced hydrogel delivery system‐based synergistic approach for tumor therapy.

## Graphical abstract

The structure of supramolecular hydrogels consists of nanomedicines (PCN‐Len nanoparticles), hydrogel backbones oxidized dextran (DX), and nanoregulators (p(Man‐IMDQ) NRs). Nanomedicine downregulates the expression of vascular endothelial growth factor, inhibits tumor angiogenesis, and reduces the density of microvessels. Nanoregulators with macrophage‐targeting ligands repolarize pro‐angiogenic M2‐type tumor‐associated macrophages TAMs to anti‐angiogenic M1‐types.

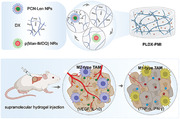

## 1 Introduction

Liver cancer is one of the most common cancers in the world and the third leading cause of cancer‐related mortality, with hepatocellular carcinoma (HCC) being the most dominant type (75–85%).^[^ ^1^ ^]^ Despite the increasing implementation of early surgery and local ablation therapy, these strategies can only cure a minority of patients diagnosed with early‐stage disease.^[^ ^2^ ^]^ Remarkable vascular abnormalities such as hypervascularity, are commonly associated with the development of HCC;^[^ ^3^ ^]^ thus, molecular therapy based on anti‐angiogenic antibodies and multi‐target tyrosine kinase inhibitors (TKI) becomes the mainstream treatment modality for most intermediate and advanced HCC in recent years.^[^ ^4^ ^]^ TKIs such as lenvatinib and sorafenib, effectively block vascular endothelial growth factor (VEGF)‐ and fibroblast growth factor (FGF)–driven angiogenesis, KIT‐dependent angiogenesis, and VEGFR3‐associated lymphangiogenesis in previous preclinical studies.^[^ ^5^ ^]^ However, multiple cell types interact with hepatocytes in the chronically inflamed liver, and several TKIs currently have limited clinical benefit.^[^ ^6^ ^]^ Infiltration of immunosuppressive cells into the treated tumor site in a metastatic HCC model develops resistance to anti‐angiogenic drug therapy after an initial antitumor response to targeted drugs within the tumor microenvironment (TME).^[^ ^7^ ^]^ Specifically, various anti‐angiogenic TKIs could induce the expression of host‐derived circulating pro‐angiogenic factors, including VEGFR2, CXCL10, SDF1, and G‐CSF, recruit pro‐angiogenic cells, and enhance tumor revascularization.^[^ ^8^ ^]^ Additionally, orally administered TKIs are generally confronted with multiple biological barriers, including drug‐degrading enzymes in stomach,^[^ ^9^ ^]^ which could decrease their therapeutic effects. Therefore, it remains a grand challenge to further optimize TKI‐based anti‐angiogenic therapy to prevent the development of HCC.

Angiogenic programming in tumor tissue is a multidimensional process regulated by cancer cells, and a variety of tumor‐associated stromal cells and pro‐angiogenic mediators, including growth factors, cytokines, various extracellular matrix proteins, and secreted extracellular vesicles.^[^ ^10^ ^]^ Compared with normal vascular endothelial cells, tumoral endothelial cells show higher proliferation, migration and tube formation capabilities,^[^ ^11^ ^]^ which significantly rely on TAMs, an important type of pro‐angiogenic cells in the TME, that could secrete growth factors and inflammatory cytokines to promote endothelial cells activation, and support angiogenesis.^[^ ^12^ ^]^ Moreover, TAMs promote the formation of vascular niche and support tumor cell dissemination to further trigger tumor cell invasiveness.^[^ ^13^ ^]^ Macrophages in tumor tissues are mainly derived from circulating monocytes, which extravasate into tumors under the action of various chemoattractants, and further differentiate into TAMs under the stimulation of macrophage colony‐stimulating factor.^[^ ^14^ ^]^ Although TAMs are generally tumor‐promoting, exploiting macrophage plasticity could switch the macrophage to antitumor phenotype. For example, toll‐like receptors (TLRs), innate immune pattern recognition receptors, have a fundamental role in the activation of innate immune responses, and the TLR7/8 ligand imidazoquinoline (IMDQ), the only TLR agonist approved for clinical use, enables the activation of TLRs, and macrophage polarization toward a pro‐inflammatory phenotype.^[^ ^15^ ^]^ Furthermore, most TAMs have the characteristics of M2 macrophages, and the mannose receptor CD206 is highly expressed on the surface of TAMs.^[^ ^16^ ^]^ Therefore, it is possible to reverse M2‐type TAMs to M1‐type through mannose‐mediated targeted strategy by improving TAMs response to subunit agonists, which is expected to reduce the vascularization induced by M2‐type TAMs and to boost TKI‐mediated anti‐angiogenesis therapy of HCC.

**Scheme 1:**
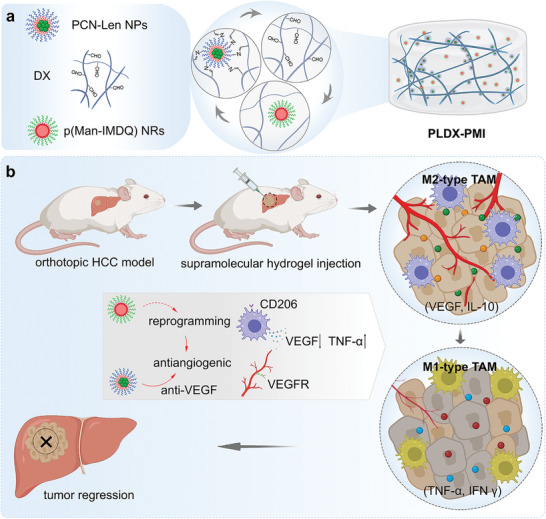
The design of TAMs‐targeted, acid‐sensitive supramolecular hydrogel for co‐delivery of TKI and TLR7/8 agonist (TLR7/8a) and its function in inducing a collaborative antitumor angiogenesis for HCC therapy. a) The structure of supramolecular hydrogels PLDX‐PMI consisting of drug‐loaded PCN‐Len NPs, hydrogel backbone chain DX, and TAMs‐targeted p(Man‐IMDQ) NRs. b) Schematic illustration of antitumor therapy toward tumor vascular network elicited by PLDX‐PMI‐mediated release of multikinase inhibitors targeting VEGFR and M2‐type TAMs repolarization. PCN‐Len NPs downregulates the expression of VEGF, inhibits tumor angiogenesis, and reduces the density of microvessels. Nanoregulators with macrophage‐targeting ligands repolarize pro‐angiogenic M2‐type TAMs to anti‐angiogenic M1‐types. This synergistically combined approach significantly enhanced the anti‐tumor angiogenesis effect of TKIs‐mediated nanomedicine in a highly malignant orthotopic HCC Hepa1‐6 model.

Herein, targeting the abnormal vascular network in the tumor microenvironment of orthotopic liver cancer, an injectable and supramolecular hydrogel system (PLDX‐PMI) co‐assembled by lenvatinib‐loaded nanomedicines (PCN‐Len NPs) with DX (denoted as PLDX) through dynamic covalent imide bonds was constructed to deliver mannose‐conjugated polyTLR7/8a nanoregulators (denoted as p(Man‐IMDQ) NRs) for tumor anti‐angiogenic therapy (**Scheme** **1**a). The prepared PLDX‐PMI supramolecular hydrogel co‐delivery system enabled the local sustained release of nanomedicines and nanoregulators in response to tumor acidic environment. As an angiogenesis inhibitor, PCN‐Len NPs targets the tyrosine kinases of vascular endothelial cells, downregulates the expression of VEGF‐A and Ang‐2, destroys angiogenesis in tumors, and reduces the density of microvessels. Self‐assembled nanoregulators with hydrophilic polymannose (macrophage‐targeting ligand) would repolarize pro‐angiogenic M2‐type TAMs into anti‐angiogenic M1‐type. Synergistically, this combination approach would significantly enhance the anti‐tumor angiogenesis effect of lenvatinib nanomedicine, as amplified by TAMs reprogramming (Scheme 1b). On the highly malignant orthotopic liver cancer Hepa1‐6 model, a single‐administration of hydrogel delivering cancer nanomedicines and macrophage polarizing nanoregulators was promising in suppressing the progression of Hepa1‐6 hepatocellular carcinoma. It is foreseeable that this hydrogel‐based synergistic therapy with significantly enhanced anti‐angiogenic efficiency may open a new avenue for cancer treatment.

## 2 Results and Discussion

### 2.1 PCN‐Len Nanomedicine Efficiently Inhibits Angiogenesis In Vitro

PCN‐Len NPs were prepared by the co‐assembly of lenvatinib and diblock copolymer PCN. PCN was synthesized by ring‐opening polymerization of *ε*‐caprolactone (*ε*‐CL) and following deprotection of tertiary butyl groups (Figure S1, Supporting Information), and characterized by ^1^H NMR (Figure S3, Supporting Information). The molecular weight ratio of hydrophilic/hydrophobic block was 1:2.9.

**Figure 1:**
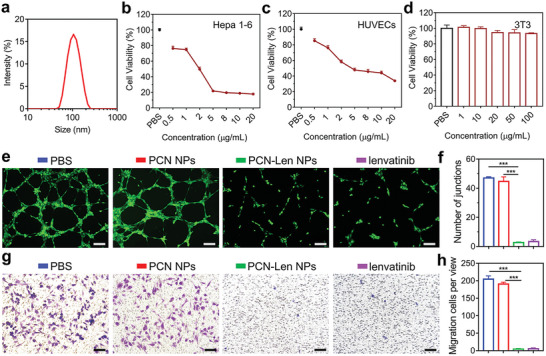
Characterization of PCN‐Len NPs, and tube formation and migration of HUVECs. a) Dynamic light scattering (DLS) profile of PCN‐Len NPs. b,c) Cytotoxicity of PCN‐Len NPs against Hepa1‐6 (b) and HUVECs (c) cells cultured with varied concentrations of lenvatinib. d) Cytotoxic effects of PCN NPs on 3T3 cells as a function of nanoparticle concentrations. e,f) Representative images and quantification of the inhibition of tubule formation of HUVECs treated with PCN‐Len NPs, lenvatinib and controls. g,h) Representative images and quantification of inhibition of HUVECs migration by transwell assay. Data represent mean ± SDs (*n* = 3). Statistical significance was analyzed using two‐tailed Student's *t*‐test between two groups. ***p < 0.001, between indicated groups.

Lenvatinib is a multi‐targeted tyrosine kinase inhibitor that inhibits vascular endothelial growth factor receptors (VEGFR1‐3), fibroblast growth factor receptors (FGFR1‐4), platelet‐derived growth factor receptors (PDGFR), stem cell factor receptor and rearrangement during transfection.^[^ ^17^ ^]^ In this study, lenvatinib was encapsulated into PCN NPs through the hydrophobic interaction between drug molecules and the hydrophobic block of PCN. The drug loading and encapsulation efficiency determined by UV–vis spectroscopy was 1.3% w/w and 65.2%, respectively (Figure S4, Supporting Information). Dynamic light scattering (DLS) analysis (**Figure** **1**a) indicated that the hydrodynamic diameter and dispersity index of PCN‐Len NPs were 116.8 ± 1.2 nm and 0.281, respectively. Also, transmission electron microscopy (TEM) image of PCN‐Len NPs showed spherical shape (Figure S5, Supporting Information). Then, PCN‐Len NPs were evaluated against tumor cells in vitro. HCC Hepa1‐6 cells were treated by PCN‐Len NPs at a concentration range of 0.5‐20 µg mL^−1^. The cell viability (Figure 1b) showed an overall decreasing trend with the increase of lenvatinib concentration and the half maximal inhibitory concentration (IC50) of PCN‐Len NPs was 1.82 µg mL^−1^. Further, as shown in Figure 1c, PCN‐Len NPs also showed an inhibitory effect on human umbilical vein endothelial cells (HUVECs) proliferation in endothelial cell medium (ECM). Moreover, PCN NPs showed no toxicity toward 3T3 cells over a concentration range of 1–100 µg mL^−1^ (Figure 1d).

Next, the anti‐angiogenic activity of PCN‐Len NPs was evaluated by VEGF‐induced tube formation and migration of HUVECs. Figure 1e shows that PBS and blank PCN NPs treatments induced tubule‐like network formation by HUVECs, while Lenvatinib and PCN‐Len NPs treatments blocked tube formation. The number of junctions in PBS and PCN NPs groups was 17.62 and 16.75 times of that in PCN‐Len NPs group, respectively (Figure 1f). HUVECs migration tested by transwell migration assays suggested the introduction of lenvatinib significantly inhibited cell migration to the lower wells (Figure 1g). The migrated cells per view in PBS and PCN NPs groups were 47.27 and 44.03 times higher than that in PCN‐Len NPs group, respectively (Figure 1h). Meanwhile, PCN NPs as the delivery carrier of lenvatinib did not affect HUVECs tube formation and migration. These data demonstrated that the PCN‐Len NPs show antitumor activity by inhibiting the migration of tube formation of HUVECs.

### 2.2 p(Man‐IMDQ) NRs Promotes Macrophage M1 Polarization In Vitro

**Figure 2:**
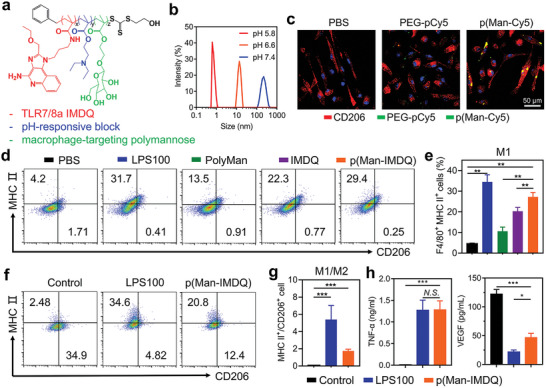
Preparation and characterization of p(Man‐IMDQ) NRs, and macrophage repolarization to M1 phenotype. a) The chemical structure of p(Man‐IMDQ). b) The average particle size distribution of p(Man‐IMDQ) NRs determined by DLS at different pH values. c) Confocal images of p(Man‐IMDQ) NRs targeting macrophages. Macrophage nucleus was stained with DAPI. d) Flow cytometry analysis of MHCII and CD206 in BMDMs with different treatments (gated on F4/80^+^ cells). e) The percentage of F4/80^+^MHCII^+^ macrophages. f) Flow cytometry analysis of MHCII and CD206 expression in BMDMs that were pre‐polarized into M2 phenotype and then stimulated with different treatments (gated on F4/80^+^ cells). g) The percentage of repolarized F4/80^+^/CD206^+^ MHCII^+^ macrophages. h) ELISA analysis of proinflammatory (TNF‐*α*) and anti‐inflammatory cytokines (VEGF) expressed by M2 phenotype macrophages. Data represent mean ± SD (*n* = 3). Statistical significance was analyzed using two‐tailed Student's *t*‐test between two groups, ***p < 0.001, **p < 0.01, and *p < 0.05.

To optimize the macrophage polarization toward a pro‐inflammatory phenotype by TLR7/8a IMDQ, p(Man‐IMDQ) was designed to comprise amphiphilic triblock copolymer chains with pH‐sensitive block, IMDQ moieties and mannose‐mediated targeting ligands (**Figure** **2**a). Subsequently, p(Man‐IMDQ) was prepared by RAFT polymerization and following replacement reaction by IMDQ (Figure S2, Supporting Information), which was characterized by ^1^H NMR (Figure S6, Supporting Information). The polymerization degree of IMDQ monomer was 7.7 (Figure S7, Supporting Information). Amphiphilic p(Man‐IMDQ) could self‐assemble into nanoscale aggregates in water, serving as nanoregulators for controlling macrophage polarization. The pH‐responsiveness of p(Man‐IMDQ) NRs was evaluated by comparing the particle size in different pH buffers (Figure 2b). Results showed that the hydrodynamic diameter of nanoparticles was 219.6 nm at pH 7.4, which was significantly decreased to 13.8 at pH 6.6. When pH value was further decreased to 5.8, p(Man‐IMDQ) nanoparticles were disassembled to polymers. In response to an acidic tumor environment, pH‐triggered disassembly of nanoaggregates can effectively enhance drug utilization efficiency.^[^ ^18^ ^]^

Next, the mannose‐mediated targeting mechanism was visualized by molecularly labeling nanoparticles with Cy5 fluorescent dye (denoted as p(Man‐Cy5) NPs) and co‐cultured with M2‐type bone marrow derived monocytes (BMDMs) with high CD206 expression, and further analyzed by CLSM. Copolymer with pH‐sensitive moiety, and PEG as the hydrophilic segment (denoted as PEG‐pCy5 NPs) was used as a control for macrophage targeting study. As shown in Figure 2c, BMDMs treated with IL‐4 displayed strong red fluorescence intensity after stained with anti‐CD206 antibody, and obvious pseudopod‐like structures. p(Man‐Cy5) NPs group shows higher colocalization with CD206 compared to PEG‐pCy5 NPs, suggesting effective mannose binding with CD206 receptors. Furthermore, flow cytometry was employed to quantify the polarization of M0‐type BMDMs into M1 phenotype by p(Man‐IMDQ) NRs. Copolymers without TLR7/8a IMDQ (denoted as PolyMan), and free IMDQ at a concentration of 10 µg mL^−1^ and LPS at a concentration of 100 µg mL^−1^ were served as controls. Figure 2d,e and Figure S8, Supporting Information, showed that p(Man‐IMDQ) NRs significantly increased the expression of MHCII, a typical marker for M1 phenotype macrophage,^[^ ^19^ ^]^ which were 22.4%, 16.63%, and 6.95% higher than that in PBS, PolyMan and IMDQ groups, respectively. Then, the macrophage repolarization induced by p(Man‐IMDQ) NRs was verified by IL‐4‐treated M2 phenotype macrophages. Results (Figure 2f,g and Figure S9, Supporting Information) indicated that p(Man‐IMDQ) NRs markedly promoted the repolarization of M2‐type macrophages into M1 phenotype (F4/80^+^ MHCII^+^ macrophages) to 20.8%, while that was 2.48% for PBS group. The ratio of M1/M2 macrophages was also significantly increased, in comparison with PBS treatment. Consistently, ELISA data (Figure 2h) showed that p(Man‐IMDQ) NRs reprogrammed the pre‐stimulated M2 type BMDMs into M1 state, as indicated by significantly increased secretion level of typical pro‐inflammatory cytokine (TNF‐*α*) and decreased production of anti‐inflammatory cytokine (VEGF). These results suggest p(Man‐IMDQ) NRs could bind to mannose receptors in macrophages, and not only prime naïve macrophages to M1 type, but also potently drive the repolarization of M2 macrophages to M1 phenotype, which are significantly better than free TLR7/8a.

### 2.3 Preparation and Characterization of p(Man‐IMDQ) NRs‐Encapsulated, Supramolecular Polymer‐Nanomedicine Hydrogel (PLDX‐PMI), and Endothelial Cell Migration and Proliferation Inhibition

**Figure 3:**
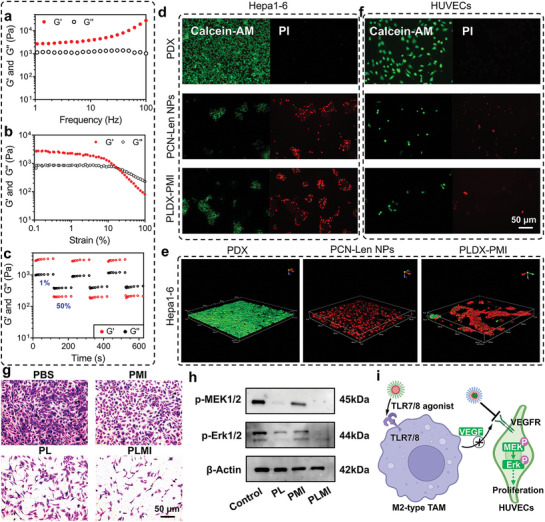
Characterization of supramolecular PLDX‐PMI hydrogel, and HUVECs migration and proliferation. a) The frequency sweep of PLDX‐PMI hydrogel detected in the angular frequency range of 1 to 100 rad s^−1^. b) The strain sweep of PLDX‐PMI hydrogel at a fixed angular frequency (1 rad s^−1^). c) The self‐healing analysis of PLDX‐PMI hydrogels. d) Representative live/dead staining and e) 3D construction of CLSM images of Hepa1‐6 cells treated by PDX, PCN‐Len NPs, and PLDX‐PMI hydrogel. f) Representative live/dead staining images of HUVECs treated by PDX, PCN‐Len NPs, and PLDX‐PMI. g) Representative images of migrated HUVECs by transwell assay. h) Protein expression level of p‐MEK1/2 and p‐Erk1/2 in HUVECs determined by western blotting. i) Schematic mechanism of PCN‐Len NPs and p(Man‐IMDQ) NRs in inhibiting endothelial cell proliferation.

Subsequently, dextran, a polysaccharide consisting of glucose,^[^ ^20^ ^]^ was selected as the backbone for hydrogel formation. Dextran was oxidized by sodium periodate to generate aldehyde groups, which cross‐linked with amino groups on the surface of PCN NPs to construct injectable hydrogels by forming imine bonds. The oxidation degree of DX analyzed by colorimetric hydroxylamine titration was 20.6% (Figure S10, Supporting Information). The mechanical properties of PLDX‐PMI hydrogels were examined by oscillatory shear rheology. For the frequency sweep, hydrogel was applied with different angular frequencies with a fixed shear strain, and the value of G′ was higher than G″ in the angular frequency range of 0.1–100 rad s^−1^, showing typical elastic solid‐like mechanical properties (**Figure** **3**a). In addition, strain sweep (Figure 3b) showed that when the strain was increased to 7.74%, the G′ value dropped remarkably, demonstrating a shear‐thinning property. At the strain of 16.9%, the value of G′ was lower than that of G″, indicating the collapse of hydrogel network, suggesting a gel‐to‐sol phase transition. The self‐healing ability of PLDX‐PMI hydrogel was further examined by step‐strain measurement under alternating high (50%) and low (1%) strains. Figure 3c showed that the values of G″ and G′ at the strain of 50% were lower than those at the strain of 1% and the value of G″ was higher than that of G′ in solution state, however, the recovery of strain to 1% enabled G′ value to be higher than G″, indicating the return to gel state. These data show PLDX‐PMI hydrogel was self‐healing and injectable. Additionally, the encapsulation of nanoregulators had no significant effect on the modulus of hydrogels (Figure S11, Supporting Information).

To investigate the pH‐sensitive drug release from PLDX hydrogels, hydrogels were cultured in PBS with pH = 7.4 or 6.5. It was found that lenvatinib exhibited a sustained pH‐responsive drug release behavior with a higher release rate at lower pH value (Figure S12, Supporting Information), which was attributed to the cleavage of imine bond at more acidic pH conditions. Cumulative drug release at day 14 was 40.1% and 79.9% at pH 7.4 and 6.5, respectively. Furthermore, the swelling rate of PLDX hydrogels at pH 6.5 or 7.4 were 37.1% and 28.7% after immersed in PBS for 12 h (Figure S13, Supporting Information). These results suggest that the supramolecular hydrogel (PDX—PCN/DX hydrogel) composed of PCN NPs and DX is promising for controllable drug delivery in response to the slightly acidic environment in tumor.

Then, the cytotoxicity of PLDX‐PMI hydrogel against Hepa1‐6 and HUVEC cells was examined. Cells were cultured in petri dishes and the hydrogel was added, and cells were stained by live/dead assay kits. As shown in Figure 3d and Figure S14, Supporting Information, a large population of alive cells (green fluorescence) with normal morphology of Hepa1‐6 cells was uniformly distributed in the petri dish containing the hydrogel in complete medium, indicating that PDX hydrogel carrier was cytocompatible. However, PCN‐Len NPs and PLDX‐PMI hydrogel show superior killing effect on Hepa1‐6 cells, as indicated by intensive red fluorescence signals, damaged cell morphology and the formation of large dead cell aggregates, which were also observed in 3D reconstruction image by CLSM (Figure 3e). The inhibition of PLDX‐PMI hydrogel on the proliferation of HUVECs was also observed by live/dead staining. Typical cobblestone cell morphology of HUVECs treated with PDX hydrogel was observed (Figure 3f and Figure S14, Supporting Information). Similar to PCN‐Len NPs treatment, the supramolecular drug‐loaded PLDX‐PMI hydrogel significantly inhibited the proliferation of HUVECs, and resulted in massive cell death, as demonstrated by spherical shape and low cell number.

Afterward, the crosstalk between macrophages and endothelial cells was investigated by transwell migration assay. p(Man‐IMDQ) NRs polarized M2‐type macrophages into M1‐type phenotypes, reduced the secretion of pro‐angiogenic factors, and inhibited the pro‐angiogenic factor‐mediated proliferation of endothelial cells. PCN‐Len NPs directly interfere with VEGF‐mediated proliferation signaling pathway in endothelial cells. Therefore, the synergistic anti‐angiogenic effect of PCN‐Len NPs and p(Man‐IMDQ) NRs was further investigated by co‐culture of macrophages and endothelial cells. BMDM‐derived macrophages were seeded in bottom chambers and subsequently stimulated by IL‐4 to produce M2 phenotype macrophages, while HUVECs were seeded in transwell insert chambers. PCN‐Len NPs and p(Man‐IMDQ) NRs were added to the medium in the upper chamber and lower chamber, respectively, and were defined as PBS group, PL group, PMI group, and PLMI group (Figure S15, Supporting Information). Figure 3g showed that compared with PBS group, PCN‐Len NPs or p(Man‐IMDQ) NRs‐treated effectively inhibited the migration of endothelial cells to the bottom chamber, and the migrating cells per view were reduced by 3.1 and 1.5 times, respectively (Figure S16, Supporting Information). Synergistically, PCN‐Len NPs combined with p(Man‐IMDQ) NRs‐treated further inhibited cell migration, and the migrating cells per view was reduced by 7.94 times. VEGF exerts pro‐angiogenic activity by binding to VEGFR with high affinity and inducing phosphorylation of VEGFR.^[^ ^21^ ^]^ Activation of phosphorylated VEGFR leads to the activation of downstream MEK/ERK cascade signaling, which plays a critical role in regulating cell survival, proliferation, and migration.^[^ ^22^ ^]^ Western blot analysis showed that p(Man‐IMDQ) NRs and PCN‐Len NPs treatment significantly downregulated the phosphorylation of MEK1/2 and Erk1/2 of HUVECs (Figure 3h and Figure S17, Supporting Information), indicating that the proliferation of HUVECs was blocked by the VEGF‐MAPK pathway (Figure 3i). These results suggested that p(Man‐IMDQ) NRs could reduce the secretion of VEGF by reprogramming M2‐type BMDMs, and combined with VEGFR‐blocking PCN‐Len NPs, and a synergistic inhibition of endothelial cell proliferation and migration was achieved.

### 2.4 Analysis of the Gene Expression Profile Dataset Related to HCC

**Figure 4:**
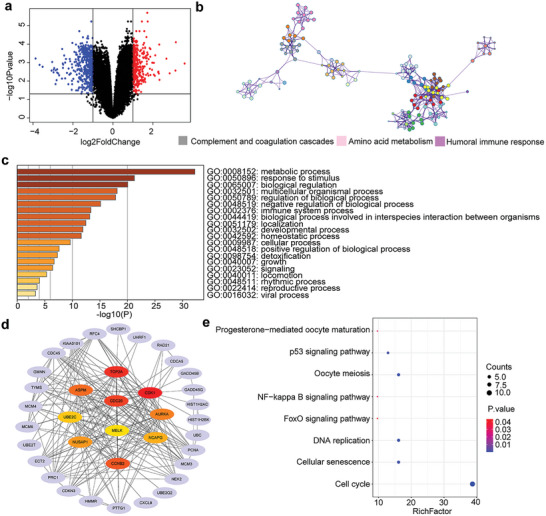
Analysis of the gene expression profile dataset related to hepatocellular carcinoma (GSE146049). a) Volcano map. The red points represent upregulated genes selected based on the corrected *p*‐value < 0.05 and logFC > 1. The blue points represent downregulated genes selected based on the corrected *p*‐value < 0.05 and logFC ←1. The black points represent genes with no significant difference. b) Enrichment GO cluster analysis of DEGs. c) Functional enrichment analysis of DEGs. d) Hub genes calculated from PPI network of DEGs by MCC algorithm. e) KEGG enrichment analysis of hub genes. FC, fold change; GO, Gene Ontology; DEGs, differentially expressed genes; KEGG, Kyoto encyclopedia of genes and genomes; PPI, protein–protein interaction.

Furthermore, “Hepatocellular carcinoma” was searched in the GEO database. Gene expression profiling dataset (GSE146049 platform: GPL10558 Illumina HumanHT‐12 V4.0 expression beadchip) was acquired, which contained five non‐tumor and five tumor biopsy samples. GEO2R online software was used to analyze datasets and find the DEGs (version 4.0.0). A total of 818 DEGs were selected from GSE146049 according to the criteria (corrected *p*‐value < 0.05 and |logFC| > 1), including 341 and 477 genes with upregulated and downregulated expressions, respectively. R language software was applied to construct volcano maps (**Figure** **4**a). Then, DAVID database (version 6.8) and Metascape (version 3.5) were chosen to perform functional and pathway enrichment analyses of screened DEGs. The enrichment GO cluster results are shown in Figure 4b, which indicated that the DEGs were remarkably enriched in immune and vascular related terms (Figures S18–S20, Supporting Information). The significant GO enrichment results shown in Figure 4c pointed that immune response and vascular system played crucial roles in the occurrence and development of hepatocellular carcinoma. In addition, the STRING database (version 11.0) and Cytoscape software (version 3.7.2) were used to construct a PPI network of DEGs. And hub genes were calculated by cytoHubba using MCC algorithm (Figure 4d). Moreover, functional enrichment of hub genes was performed. Eight key pathways were obtained (Figure 4e), some of which related to inflammation and cell proliferation (Figure S21, Supporting Information). To sum up, the DEGs functional enrichment analysis confirmed that inflammatory response and vascular system played crucial roles in the occurrence and development of hepatocellular carcinoma, which matched the aforementioned in vitro results, as well as provided the bioinformatics evidence of the rational design of the hydrogel system.

### 2.5 Antitumor Activity of PLDX‐PMI Hydrogel toward HCC

**Figure 5:**
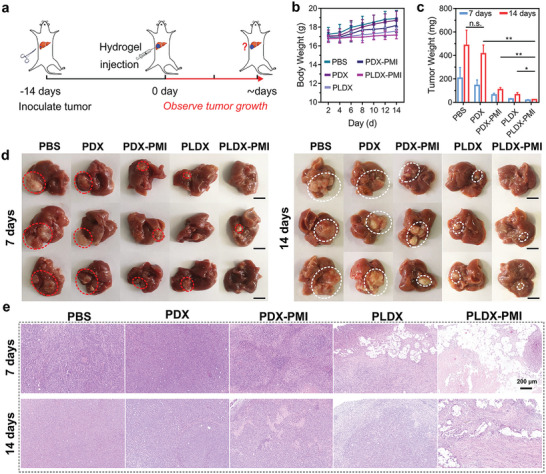
In vivo inhibition of orthotopic HCC progression by enhanced anti‐angiogenic therapy via co‐delivery of PCN‐Len NPs and p(Man‐IMDQ) NRs in supramolecular hydrogel. a) Schedule for orthotopic Hepa1‐6 HCC treatment. b) Curves of mouse body weight. Data represent mean ± SD (*n* = 5). c) The weight of tumors at the 7th and 14th day after treatment. Data represent mean ± SD (*n* = 3). d) Photographs of resected tumor‐bearing livers after treatment for 7 and 14 days. e) Representative images for H&E staining of tumor tissue sections. Statistical significance was analyzed using two‐tailed Student's *t*‐test between two groups, **p <0.01 and *p <0.05.

Next, in vivo degradation of PLDX hydrogels was first investigated by non‐invasive fluorescence imaging. PLDX labeled with Cy5 was injected subcutaneously into the back of C57BL/6 mice, and fluorescent images were recorded at predetermined time points (Figure S22, Supporting Information). The fluorescence intensity was decreased gradually with time, suggesting the degradation of hydrogel, and after 10 days, the reduction rate of fluorescence intensity was 81.6% compared with the initial hydrogel. Furthermore, based on the synergistic strategy of effective vascular disruption by PCN‐Len NPs directly and M2 macrophage‐repolarizing p(Man‐IMDQ) NRs indirectly, therapeutic tumor inhibition efficiency was evaluated in orthotopic HCC tumor model. Hepa1‐6 tumor tissue blocks were transplanted into the liver of C57BL/6 mice to construct a mouse model of transplanted HCC. The treatment and monitoring protocol was shown in **Figure** **5**a.

Mice were randomly divided into five groups and received in situ injection of various formulations including PBS, blank PDX hydrogel, p(Man‐IMDQ) NRs encapsulated in PCN/DX (PDX‐PMI) hydrogel, PLDX hydrogel, and PLDX‐PMI hydrogel at a volume of 100 µL. The dose of lenvatinib and IMDQ was 10 and 0.5 mg kg^−1^, respectively. No significant reduction in animal body weight (Figure 5b) was observed during the time range of 14 days. Moreover, PLDX‐PMI treatment effectively prolonged the long‐term survival of mice. On the 30th day, the survival rate of tumor‐bearing mice treated with PLDX‐PMI was 90%, while that in PBS group was only 20% (Figure S23, Supporting Information). On the 7th and 14th day, tumor‐bearing mice were sacrificed, and liver tissues were excised, weighed, and photographed. As shown in Figure 5c, the weight of orthotopic liver tumors resected at the 14th day shows that the local tumor burden of combination treatment group was only 23.98 ± 2.47 mg, which was significantly decreased by 95.1% and 94.1%, in comparison with that of PBS (486.4 ± 129.3 mg) and PDX group (415.1 ± 73.8 mg), respectively. In contrast, the reduction rate of tumor burden for PDX‐PMI or PLDX treatment was 77.8% and 63.3%, respectively, compared with the administration of PBS. As shown in Figure 5d, large tumors appeared in both the control and PDX hydrogel groups, while the tumor size was significantly reduced in PDX‐PMI or PLDX group. Tumors were even completely regressed with the treatment of PLDX‐PMI hydrogel.

Then, tumor tissues were sliced and stained with hematoxylin and eosin (H&E) for further analyzing the therapeutic effects. As shown in Figure 5e, typical tumor pathological structures and a large number of tumor cells were observed in liver tumors received the injection of PBS or PDX hydrogel. Compared with PBS group, the monotherapy resulted in extensive cancer cell apoptosis, while the combination therapy of PLDX‐PMI hydrogel induced an obviously higher level of Hepa1‐6 cell apoptosis. Quantification assessment revealed PLDX‐PMI resulted in a significantly higher percentage of necrotic tumor tissue (68.69%), while it was 8.62%, 9.95%, 28.05%, and 49.12% (Figure S24, Supporting Information) for PBS, PDX, PDX‐PMI, and PLDX group, respectively. These results suggest that synergistic anti‐angiogenesis therapy can significantly suppress the growth of malignant orthotopic HCC. Histological staining of major organs including heart, spleen, lung, and kidney showed that all treatments did not induce obvious inflammatory cell infiltration and pathological changes (Figure S25, Supporting Information), indicating a good biocompatibility of supramolecular hydrogels in vivo.

### 2.6 Anti‐Angiogenesis and TAMs Repolarization Induced by PDLX‐PMI Hydrogel in HCC Therapy

**Figure 6:**
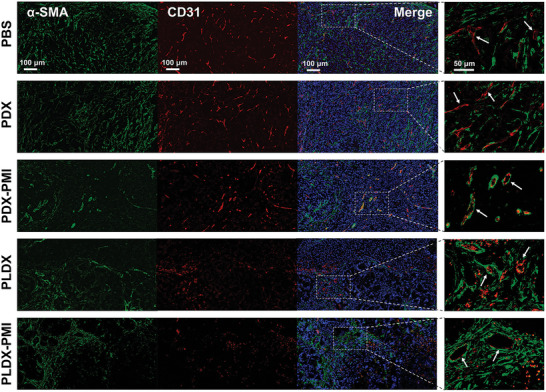
PCN‐Len NPs and p(Man‐IMDQ) NRs effectively decreased blood vessel density in HCC tumor tissues. Representative images of orthotopic Hepa1‐6 liver cancer tissue vasculature stained with CD31 (red) and *α*‐SMA (green), while nuclei were stained with DAPI.

Anti‐angiogenesis was further investigated by analyzing the vascular density in tumor tissue. Blood vessels were stained with anti‐CD31 antibody, a typical marker for vascular endothelial cells. Representative immunofluorescence images demonstrated the production of tumor vasculature at day 7 for different treatment groups (**Figure** **6**). Tumors in PBS and PDX groups showed a higher density of vascular areas and abnormal vascular structures. CD31‐positive vascular area in PLDX‐PMI group was reduced by 7.2, 6.56, 2.81, and 1.19 times at the 7th day, compared with that in PBS, PDX, PDX‐PMI, and PLDX group, respectively (Figure S26, Supporting Information). Monotherapy with PDX‐PMI or PLDX hydrogel system containing TLR7/8a or VEGFR inhibitor nanoparticles also significantly decreased the tumor vessel density, in comparison with PBS treatment.

Further, the vascular maturation of liver cancer tissues was stained by anti‐*α*‐SMA antibody, a surface marker for smooth muscle cells and cancer‐associated fibroblasts.^[^ ^23^ ^]^ Inhibition of angiogenesis is often associated with changes in vascular maturation,^[^ ^24^ ^]^ and normalization of the vasculature can improve oxygen levels, drug delivery, and immune cell infiltration.^[^ ^25^ ^]^ Indicated by both *α*‐SMA^+^ and CD31^+^ cells, tumor blood vessels in PBS and PDX group were poorly covered by vascular smooth muscle cells (Figure 6). However, blood vessels in tumors treated by mono‐ or combinational therapy were distinctly surrounded by vascular smooth muscle cells. The percentage of smooth muscle cell coverage on blood vessels in PDX, PDX‐PMI, PLDX, and PLDX‐PMI group was 60.5%, 93.21%, 89.15%, and 97.18%, respectively (Figure S26, Supporting Information), by calculating the ratio of CD31^+^ *α*‐SMA^+^ cells to CD31^+^ cells alone. These results suggest the treatment of macrophage manipulation, VEGFR inhibition or combination approach could effectively induce the normalization of tumor blood vessels. These findings indicated that anti‐angiogenesis was associated with antitumor growth efficiency, and the treatment of PDLX‐PMI hydrogel tremendously reduced the microvessel density in tumor tissue and promoted the maturation of tumor blood vessels.

**Figure 7:**
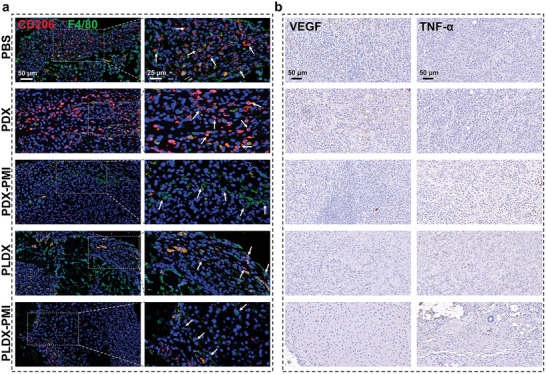
PCN‐Len NPs and p(Man‐IMDQ) NRs induced M2‐type TAM reprogramming and promoted anti‐angiogenesis. a) Representative images of CD206 (red)/F4/80 (green) immunofluorescence staining of tumor tissues. b) Representative IHC images of VEGF and TNF‐*α* expressions in tumor sections from PBS, PDX, PDX‐PMI, PLDX, and PLDX‐PMI‐treated mice.

Tumor cells construct a TME to manipulate TAMs toward pro‐angiogenic M2 type. To further confirm the role of macrophage phenotype in anti‐angiogenesis, macrophages in tumor tissues were stained with anti‐F4/80 and anti‐CD206 antibodies, for labeling total macrophages and M2‐type macrophages, respectively. Immunofluorescence images showed that the infiltration of F4/80^+^CD206^+^ macrophages (**Figure** **7**a) was notably decreased and the percentage of F4/80^+^CD206^+^ macrophages was lower in PDX‐PMI group than that in PLDX group, suggesting the effectiveness of TLR7/8a in directly polarizing M2‐type TAMs. The overlapping ratios of CD206 and F4/80 signals (Figure S27, Supporting Information) in the PBS, PDX, PDX‐PMI, and PLDX groups were 14.61, 16.41, 2.17, and 8.65 times of that in the PLDX‐PMI group, respectively. Furthermore, the infiltration of CD68^+^CD86^+^ macrophages (Figure S28, Supporting Information) was significantly increased with the treatment of PLDX‐PMI hydrogel, in comparison with PBS or PLDX, suggesting the augmentation of M1 macrophages in tumor tissue.

Combined with characterization of blood vessels and macrophages in tumor tissue by immunofluorescence detection, PLDX‐PMI treatment was associated with lower vascular network density, higher pericyte coverage on blood vessels, and lower M2‐type TAMs infiltration. Compared with tissue vessels, tumor vessels usually consist of a single endothelial layer,^[^ ^26^ ^]^ exhibit tortuous and disordered vascular morphology, and a high degree of vascular heterogeneity.^[^ ^27^ ^]^ TKIs can inhibit the formation of new blood vessels to reduce nutrient supply, however, previous studies have shown that TKIs can deplete activated pericytes, resulting in vascular leakage.^[^ ^10^, ^28^ ^]^ Therefore, the introduced macrophage‐reprogramming nanoregulator modulates vascular pericytes during angiogenesis by regulating the macrophage phenotype to block the expression of cytokines such as VEGF‐A, which induces the formation of a VEGFR‐2/platelet‐derived growth factor receptor‐beta (PDGFR‐*β*) complex, inhibiting pericyte recruitment and vascular maturation.^[^ ^29^ ^]^ Thereby enhancing adhesion between pericytes and endothelial cells, reducing vascular permeability, inhibiting tumor cell invasion, promoting tumor perfusion, and improving drug treatment efficiency.

Furthermore, representative immunohistochemistry (IHC) images demonstrated excessive VEGF expression was observed in PBS and PDX‐treated tumor tissues at day 7 (Figure 7b and Figure S29, Supporting Information). Nevertheless, PLDX‐PMI treatment markedly decreased VEGF secretion in tumor, compared with PDX‐PMI or PLDX. Also, repolarizing TAMs toward a proinflammatory state is commonly associated with an increased expression of mediators such as tumor necrosis factor *α* (TNF‐*α*), which plays a significant role at the effector stage of the antitumor response.^[^ ^30^ ^]^ Representative IHC images (Figure 7b and Figure S29, Supporting Information) demonstrated TNF‐*α* protein expression was significantly increased in HCC tissue from mice treated with TLR7/8a and VEGFR inhibitor. These evidences suggested that PDLX‐PMI hydrogel could not only reduce the number of tumor‐infiltrating macrophages but also polarize TAMs to anti‐angiogenic phenotype, exerting superior antitumor effect against HCC in combination with TKIs inhibition.

## 3 Conclusion

In summary, we demonstrated a novel synergistic combination strategy for augmented anti‐tumor angiogenesis therapy in HCC. Co‐delivery of lenvatinib nanomedicines and macrophage‐priming polyTLR7/8a nanoregulators in supramolecular dextran‐nanomedicine hydrogel can effectively reduce the microvessel density in tumor tissue, promote the normalization and pericyte coverage of tumor vessels, and stimulate the polarization of TAMs into anti‐angiogenic M1 phenotype, thus overcoming the current limitation of TKIs for antivascular therapy. Importantly, a single injection of the supramolecular hydrogel formulation at orthotopic HCC tumors significantly inhibited the tumor progression compared with hydrogel delivering nanomedicines or nanoregulators alone. The present study offers an efficient and promising strategy toward orthotopic HCC therapy by combining nanomedicine‐induced blockade of VEGF pathway in endothelial cells with nanoregulators‐mediated TAMs polarization in TME. Supramolecular polymer‐nanomedicine hydrogel also provides a versatile platform for co‐delivery of functional nanotherapeutics to treat cancer and infectious diseases.

## 4 Experimental Section

### Materials

Information for materials, synthesis and characterization of PCN, p(Man‐IMDQ) and DX, cell lines, and animals are shown in the Supporting Information. All animal procedures were reviewed and ethically approved by Chinese Academy of Medical Sciences Institute of Radiation Medicine, Animal Experiment Ethics Committee (Approval No: SYXK (Jin) 2019‐0002).

### Preparation and Characterization of Lenvatinib‐Loaded PCN Nanoparticles

Lenvatinib‐loaded PCN nanoparticles (PCN‐Len NPs) were prepared by co‐assembly method. Briefly, lenvatinib (5 mg) and PCN (500 mg) were dissolved in DMSO (3 mL) and then added dropwise into deionized water (50 mL) with vigorous stirring. Subsequently, were dialyzed with deionized water, and the volume of deionized water was adjusted to finally obtain the formulation of nanoparticles. To determine the drug loading amount and encapsulation efficiency, the nanoparticle solution was centrifuged and the absorbance of supernatant was determined by ultraviolet–visible spectrophotometer. The size of PCN‐Len NPs was determined via DLS (Zetasizer Nano ZS, Malvern). The shape and morphology of PCN‐Len NPs were assessed by TEM.

### Preparation and Characterization of p(Man‐IMDQ) Nanoregulators

p(Man‐IMDQ) nanoregulators (p(Man‐IMDQ) NRs) were prepared by the method reported in the previous works.^[^ ^31^ ^]^ Briefly, the polymer (10 mg) was dissolved in DMSO (1 mL) and then added dropwise into deionized water (10 mL) with vigorous stirring. With the evaporation of the solvent, the volume of deionized water was adjusted to finally obtain the formulation of nanoparticles with a concentration of 1 mg mL^−1^. The size of p(Man‐IMDQ) NRs were determined via Zetasizer Nano ZS (Malvern).

### Preparation of PEG‐Poly(2‐(Diethylamino)ethyl Methacrylate‐co‐Cy5) and Poly(2‐Methacryloyloxyethyl‐d‐mannoside‐co‐2‐(Diethylamino)ethyl Methacrylate‐co‐Cy5) (p(Man‐Cy5))

PEG‐poly(2‐(diethylamino)ethyl methacrylate‐co‐Cy5) (PEG‐pCy5) was synthesized by two steps. First, mPEG‐based macro‐RAFT agent PEG2000‐CTAm (200 mg, 0.085 mmol), DEAEMA (788 mg, 4.25 mmol), and methacrylic acid pentafluorophenyl ester monomer (101 mg, 0.43 mmol) were dissolved in DMF (4 mL) with stirring, and then AIBN initiator was added. After polymerization for 24 h at 70 °C under argon protection, the resultant solution was dialyzed against deionized water and lyophilized to obtain copolymer PEG‐poly(2‐(diethylamino)ethyl methacrylate‐co‐methacrylic acid pentafluorophenyl ester). Next, the resultant copolymer, NH_2_‐Cy5 and TEA were dissolved in DMF with stirring. After reaction for 96 h at 40 °C, the solvent was dialyzed against deionized water and lyophilized to obtain copolymer PEG‐pCy5.

Poly(2‐methacryloyloxyethyl‐d‐mannoside‐co‐2‐(diethylamino)ethyl methacrylate‐co‐Cy5) (p(Man‐Cy5)) was synthesized by three steps. First, the RAFT agent BHCT (4.2 mg, 0.017 mmol), DEAEMA (0.158 mg, 0.85 mmol), and methacrylic acid pentafluorophenyl ester monomer (40.7 mg, 0.17 mmol) were dissolved in DMF (1.2 mL) with stirring, and then AIBN initiator was added. After polymerization for 24 h at 70 °C under argon protection, 2‐methacryloyloxyethyl‐d‐mannoside monomer (100 mg, 0.34 mmol) was further added to continue the polymerization for 24 h. The resultant solution was dialyzed against deionized water and lyophilized to obtain copolymer poly(2‐methacryloyloxyethyl‐d‐mannoside‐co‐2‐(diethylamino)ethyl methacrylate‐co‐methacrylic acid pentafluorophenyl ester). Next, copolymer, NH_2_‐Cy5 and TEA were dissolved in DMF with stirring. After reaction for 96 h at 40 °C, the solvent was dialyzed against deionized water and lyophilized to obtain copolymer p(Man‐Cy5).

### Preparation and Rheological Analysis of Acid‐Sensitive Supramolecular Hydrogels

Acid‐sensitive supramolecular hydrogels were formed through the formation of imine bonds by mixing the DX solution (10 wt%) with PCN‐Len NPs solution (20 wt%). Gelation occurs quickly once vortexed.

The rheological analysis of acid‐sensitive supramolecular hydrogels was performed on Anton Paar (MCR 302) rheometer. Storage modulus (G′) and elastic modulus (G″) were determined by increasing the angular frequency and the shear strain of 1%. The strain sweep was examined by increasing the shear strain and the angular frequency of 1%. The self‐healing properties of the supramolecular hydrogel were also investigated by monitoring the changes of G′ and G″ under continuous train sweep with an alternative large oscillation force (50%) and a small one (1%).

### Cytotoxicity Evaluation of PCN‐Len NPs

The toxicity of PCN NPs was evaluated by CCK‐8 assay. 100 µL of 3T3 cells suspension in DMEM medium supplemented with 10% FBS was placed in 96‐well plates and incubated at 37 °C in an incubator for 24 h under humidified conditions of 5% CO_2_. Then, the culture medium was discarded, and 10 µL test substance and 90 µL of fresh medium were added to the culture plate. Three parallel groups were given. Cells were further cultured for 24 h, and then 10 µL of CCK‐8 solution was added to each well and incubated for additional 4 h. The absorbance was determined using a Varioskan Flash (Thermo Scientific) at a wavelength of 450 nm. The cell viability was calculated by comparing the absorbance value from treatment group with that of the control group.

Further, the cytotoxicity of PCN‐Len NPs to HUVECs and Hepa1‐6 cells were evaluated by CCK‐8 assay. 1 × 10^4^ HUVECs or Hepa1‐6 cells were cultured in ECM or DMEM complete medium containing 10% FBS in 96‐well plates for 24 h. The medium was removed, and fresh medium containing PCN‐Len NPs was added. The concentration of lenvatinib in the medium was 0.5, 1, 2, 5, 8, 10, and 20 µg mL^−1^. Further, cell killing was assessed by CCK‐8 assay according to manufacturer's guideline.

### Cytotoxicity Evaluation of Supramolecular Hydrogels

The cytotoxicity of supramolecular hydrogels was evaluated by live/dead staining. 500 µL of 5 × 10^4^ HUVECs or Hepa1‐6 cells suspension in ECM or DMEM complete medium was placed in 24‐well plates and incubated at 37 °C in an incubator for 24 h under humidified conditions of 5% CO_2_. Further, blank supramolecular hydrogels or lenvatinib‐loaded supramolecular hydrogels containing 10 µg mL^−1^ lenvatinib were added to the culture medium. After another 24 h of incubation, the media were replaced with fresh media, Calcein‐AM and propidium iodide (Invitrogen USA) were used for live/dead staining according to the manufacturer's steps.

### In Vitro Assessment of Endothelial Cell Migration and Tube Formation

For cell migration assay, 1 × 10^4^ HUVECs suspended in ECM was placed in the upper chamber of a 12‐well transwell and incubated at 37 °C in an incubator under humidified conditions of 5% CO_2_. The lower chamber was added with 100 µL PCN NPs or PCN‐Len NPs. After incubation for 24 h, the migrated cells at the bottom of the filter were fixed with 4% paraformaldehyde, and then stained with crystal violet solution for 30 min, and the number of migrated cells was further photographed and counted under an optical microscope (Leica, Germany).

For tube formation assay, thawed Matrigel (BD, USA) was added into a pre‐cooled 24‐well plate and incubated for 30 min at 37 °C. Then, HUVECs suspension (cell number, 5 × 10^4^) were seeded on the gel‐coated plate and incubated for 24 h to form tubule‐like network. Then, the cell supernatant ECM was replaced by ECM containing PCN NPs or PCN‐Len NPs solution and cultured for 24 h. Afterward, HUVECs were stained with Calcein‐AM and visualized by CLSM, and tube formation density was quantified using ImageJ software.

### In Vitro Assessment of Macrophage Targeting by p(Man‐IMDQ) NRs

To visualize mannose targeting M2 BMDM, 2 × 10^5^ M0 BMDM were seeded on confocal dishes overnight while IL‐4 was added to induce M2 BMDM polarization. Thereafter, the PEG‐pCy5 NPs and p(Man‐Cy5) NPs solution was added and cultured for 8 h. Subsequently, the cells were washed twice with PBS, and CD206 was stained with APC‐conjugated anti‐CD206 antibodies overnight. The cell nucleus was then stained with 4′,6‐diamino‐2‐phenylindole (DAPI) (Solarbio, Catalog: C0065). Cells were washed three times with PBS and visualized by confocal microscopy (ZEISS, LSM710).

### In Vitro Assessment of p(Man‐IMDQ) NRs on Macrophage Polarization

The extraction and culture of BMDMs were conducted according to previously reported procedure.^[^ ^32^ ^]^ Then, 1.5 mL of 4 × 10^5^ BMDM suspension in medium was placed in 12‐well plates and incubated at 37 °C in an incubator under humidified conditions of 5% CO_2_. Different concentrations of LPS, IMDQ, or p(Man‐IMDQ) NRs were added to the well plates to induce the polarization of BMDM. PBS‐treated cells were used as the control. Then, cells were first blocked with anti‐mouse CD16/32 antibody for 15 min at 4 °C, stained with anti‐mouse F4/80, MHCII, CD206 antibodies and washed with PBS for three times. The prepared cell suspension was analyzed by flow cytometry (BD Accuri C6).

For macrophage repolarization study, 1.5 mL of 4 × 10^5^ BMDM suspension was placed in 12‐well plates and pre‐induced into a M2 phenotype by treatment with IL‐4 (40 ng mL^−1^) for 48 h. The cells were further stimulated with p(Man‐IMDQ) NRs (10 µg mL^−1^ IMDQ) for 24 h. Then, cells were blocked with anti‐mouse CD16/32 antibody for 15 min at 4 °C, stained with anti‐mouse F4/80, MHCII, CD206 antibodies and washed with PBS for three times. The prepared cell suspension was analyzed by flow cytometry (BD Accuri C6). Cell culture media were taken out for detecting the concentration of various secreted cytokines by ELISA kits according to the manufacturer's steps.

### In Vitro Endothelial Cell Proliferation

For the assay of endothelial cell proliferation inhibited by p(Man‐IMDQ) NRs and PCN‐Len NPs, a transwell co‐culture system of BMDM and HUVECs was used. 1 × 10^5^ HUVECs suspension was placed in the upper chamber of a 6‐well transwell. 5 × 10^5^ M2‐type BMDM suspensions were placed in the lower chamber of a 6‐well transwell, and cultured in an incubator at 37 °C under 5% CO_2_ humidified conditions. In addition, PCN‐Len NPs (lenvatinib concentration, 10 µg mL^−1^) and p(Man‐IMDQ) NRs (IMDQ concentration, 10 µg mL^−1^) were added to the upper and lower chambers of the 6‐well transwell, respectively. After incubation for 24 h, the migrated HUVECs at the bottom were fixed with 4% paraformaldehyde, stained with crystal violet solution for 30 min, and observed by an optical microscope (Leica, Germany).

### Western Blot

Western blot analysis of extracts from HUVECs with p‐MEK 1/2 antibody or p‐Erk 1/2 antibody, and detailed process are shown in the Supporting Information.

### In Vivo Inhibition of Tumor Growth of HCC

Mouse model of orthotopic Hepa1‐6 liver cancer was constructed. 1 × 10^6^ Hepa1‐6 cells were subcutaneously transplanted into the right armpit of C57BL/6 mice. When the tumor volume reached about 500 mm^3^, the tumor was excised and cut into small pieces (20 mm^3^) and inoculated into the liver tissue of recipient C57BL/6 mice. After growing for 2 weeks in the liver, mice were randomly divided into five groups with ten mice each, and were injected in situ immediately at the location of orthotopic HCC tumors with a single administration of PBS, blank PDX, PDX‐PMI, PCN‐Len/DX hydrogel (PLDX), or p(Man‐IMDQ) NRs encapsulated in PCN‐Len/DX hydrogel (PLDX‐PMI). Mice were sacrificed on the 7th and 14th day post treatment, and the tumor‐bearing livers were photographed and weighed. The animal body weight was also recorded every 2 days.

### Tumor Tissue Histology and Immunofluorescence Analysis

For tissue section staining, tumors were fixed in 4% paraformaldehyde solution for 24 h, and then dehydrated, embedded, sectioned, and stained with H&E, immunofluorescence (IF), or IHC antibody, according the manufacturer's instruction. The slice thickness was 5 µm.

For IF staining of tumor tissue vessels, the sections were immersed in boiling citrate solution and then covered by primary antibody solutions of CD31 and *α*‐SMA. For IF staining of tumor tissue macrophages, the sections were immersed in boiling citrate solution and then covered by primary antibody solutions of CD206 and F4/80 or CD68 and CD86. The fluorescein isothiocyanate‐conjugated secondary antibody was used to incubate the sections. The nuclei were counterstained by DAPI. Stained sections were observed by CLSM, and the semi‐quantitative analyses were determined using ImageJ software.

For IHC staining of cytokine expression in tumor tissue, sections were immersed in citrate buffer and boiled, then incubated overnight at 4 °C with the corresponding anti‐VEGF or anti‐TNF‐*α* antibodies. Conjugate with HRP‐conjugated secondary antibody at 37 °C for 30 min, and then treated with 3,3′‐diaminobenzidine staining. All the above sections were examined by light microscope.

### In Vivo Biosafety Evaluation

For the evaluation of systemic toxicity of the treatment strategy, on the 14th day of treatment, major organs including heart, spleen, lung, and kidney were collected, fixed, dehydrated, embedded, sectioned, and stained with H&E.

### Statistical Analysis

All data were presented as mean ± standard deviations (SDs). Differences between two groups were evaluated by two‐tailed Student's *t*‐test in Graphpad Prism. *p* < 0.05 was considered statistically significant. n.s., not significant.

## Conflict of Interest

The authors declare no conflict of interest.

## Supporting information

Supporting Information

## Data Availability

Research data are not shared.
