# Supplementary material for: Supramolecular Polymer‐Nanomedicine Hydrogel Loaded with Tumor Associated Macrophage‐Reprogramming polyTLR7/8a Nanoregulator for Enhanced Anti‐Angiogenesis Therapy of Orthotopic Hepatocellular Carcinoma
Source: Adv Sci (Weinh). 2023 May 25;10(22):2300637. doi: 10.1002/advs.202300637 (PMC10401096; doi:10.1002/advs.202300637)
Supplement: Supplementary file 1 — Supporting Information [file ADVS-10-2300637-s001.pdf]

ADVANCED SCIENCE
Open Access

Supporting Information

for *Adv. Sci.*, DOI 10.1002/advs.202300637

Supramolecular Polymer-Nanomedicine Hydrogel Loaded with Tumor Associated Macrophage-Reprogramming polyTLR7/8a Nanoregulator for Enhanced Anti-Angiogenesis Therapy of Orthotopic Hepatocellular Carcinoma

Xiang Liu, Yini Huangfu, Jingrong Wang, Pengxu Kong, Weijun Tian, Peng Liu, Chuang Fang, Shuangyang Li, Yu Nie, Zujian Feng, Pingsheng Huang, Shengbin Shi, Chuangnian Zhang, Anjie Dong* and Weiwei Wang**

Supporting Information

Supramolecular polymer-nanomedicine hydrogel loaded with tumor associated macrophage-reprogramming polyTLR7/8a nanoregulator for enhanced anti-angiogenesis therapy of orthotopic hepatocellular carcinoma

Xiang Liu, Yini Huangfu, Jingrong Wang, Pengxu Kong, Weijun Tian, Peng Liu, Chuang Fang, Shuangyang Li, Yu Nie, Zujian Feng, Pingsheng Huang, Shengbin Shi, Chuangnian Zhang, Anjie Dong*, Weiwei Wang**

Methods

Chemical regents. Sodium periodate, dextran, poly(ethylene glycol) (PEG, Mn=2000 g moL^{-1}), 2-(Diethylamino)ethyl methacrylate (DEAEMA), pentafluorophenyl 2-methylprop-2-enoate (PFPMA), ε-caprolactone, stannous octoate, trifluoroacetic acid, azobisisobutyronitrile (AIBN), trifluoroacetic acid (TFA) and triethylamine (TEA) were purchased from Sigma-Aldrich. Butyloxycarbonylamino-hydroxy poly(ethylene glycol) (Boc-NH-PEG-OH) (PEG, Mn=1500 g moL^{-1}) and NH_2-Cy5 were provided by Bankpeptide Biological Technology Co., LTD (Hefei, China). RAFT agent (2-benzylsulfanyl thiocarbonylsulfanyl)ethanol (BHCT) was synthesized according to the previously reported method.[1] RAFT agent PEG2000-CTAm was synthesized also according to the previously reported method.[2] Dichloromethane (DCM), N,N-dimethylformamide (DMF), dimethyl sulfoxide (DMSO) and diethyl ether were purchased from Jiangtian company (Tianjin, China). 2-Methacryloyloxyethyl-D-mannoside was provided by Meikai Technology Co., LTD (Shanghai, China). Imidazoquinoline was purchased from InvivoGen (San Diego CA, USA). Lenvatinib was purchased from MedChemExpress (New Jersey, USA). Mouse TNF-α (Catalog: SEKM-0034) ELISA kits and Mouse VEGF (Catalog: SEKM-0039) ELISA kits were received from Solarbio company (Beijing, China).

Antibodies including anti-mouse F4/80-PE (Clone: BM8, Catalog: 123110), anti-mouse MHC II-FITC (Clone: 10-3.6, Catalog: 109905), anti-mouse CD206-APC (Clone: C068C2, Catalog: 141708) and anti-mouse CD16/32-TruStain FcX™ (Clone: 93, Catalog: 101320) were provided by BioLegend (San Diego, California, United States).

Cell lines and Animals. HUVECs, Hepa 1-6 cells, 3T3 cells were purchased from the Cell Bank of China Academy of Sciences, and cultured according to the manufacture's guidelines. Briefly, Hepa 1-6 cells were maintained in high glucose DMEM medium supplemented with 10% FBS, 100 U ml^{-1} penicillin and 100 U ml^{-1} streptomycin and cultured at 37 ^{o}C, with a humidity of 70% - 80%, and in an environment containing 5% CO_2. HUVECs were maintained in Endothelial Cell Medium and cultured at 37 ^{o}C, with a humidity of 70% - 80%, and in an environment containing 5% CO_2.

C57BL/6 (6-8 weeks) were purchased from Vital River Laboratory (Beijing, China) and provided by Caner Hospital, Chinese Academy of Medical Sciences, respectively. All animal procedures were reviewed and ethically approved by Chinese Academy of Medical Sciences Institute of Radiation Medicine, Animal Experiment Ethics Committee (Approval No: SYXK (Jin) 2019-0002).

Synthesis of Poly(ε-caprolactone)-poly(ethylene glycol)-NH_2 (PCN). As shown in Figure S1, poly(ε-caprolactone)-poly(ethylene glycol) was synthesized by initiating ring-opening polymerization of ε-caprolactone (CL) using Boc-NH-PEG-OH as an initiator and stannous octoate as a catalyst. Boc-NH-PEG-OH and CL at pre-determined molar ratios were added in the tube and after degassing and polymerization at 130 ^{o}C for 12 h under continuous stirring, products were precipitated in cold diethyl ether. Further, the obtained copolymer was dissolved in dichloromethane, and trifluoroacetic acid was added to remove the Boc group, dialyzed against deionized water, and lyophilized to obtain PCN.

Synthesis of poly(2-Methacryloyloxyethyl-D-mannoside -*co*- 2-(Diethylamino)ethyl methacrylate -*co*- IMDQ-prop-2-enoate) (p(Man-IMDQ)). p(Man-IMDQ) was synthesized by three steps (Figure S2). First, the RAFT agent BHCT (4.2 mg, 0.017 mmol), DEAEMA (158 mg, 0.85 mmol) and PFPMA (100 mg, 0.17 mmol) were dissolved in DMF (1.2 mL) with stirring, and then AIBN initiator was added. After polymerization for 24 h at 70 ^{o}C under argon protection, 2- methacryloyloxyethyl-D-mannoside monomer (292 mg, 0.34 mmol) was added and the polymerization was continued for 24 h. The resultant solution was dialyzed against deionized water and lyophilized to obtain copolymer poly(2-Methacryloyloxyethyl-D-mannoside -*co*- 2-(Diethylamino)ethyl methacrylate -*co*- pentafluorophenyl 2-methylprop-2-enoate). Next, the prepared copolymer (100.0 mg, 0.006 mmol), imidazoquinoline (IMDQ) (66.4 mg, 0.22 mmol) and TEA (67.3 mg, 0.67 mmol) were dissolved in DMF (2 mL) with stirring. After reaction for 96 h at 40 ^{o}C, the solvent was dialyzed against deionized water and lyophilized to obtain copolymer p(Man-IMDQ). The chemical structure of p(Man-IMDQ) was determined by ^{1}H NMR spectra.

Synthesis of oxidized dextran (DX). DX was synthesized by oxidation reaction. Briefly, dextran (1 g, 2% w/v) was dissolved in deionized water, and the calculated amount of sodium periodate in aqueous solution was added dropwise with stirring overnight at room temperature in dark. The reaction was quenched by adding excess amount of ethylene glycol and dialyzed against water and lyophilized to obtain DX. The degree of oxidization was determined by colorimetric analysis.

Western Blot. HUVECs lysates were lysed by RIPA lysis buffer (Beyotime, P0013B) containing 1 mM PMSF (Beyotime, ST506). After incubation for 30 min on ice, the supernatant was collected after centrifugation. Then protein was denatured at 100 ^{o}C for 10

min. After mixing with loading buffer containing bromophenol blue, protein samples were separated by a 10% sodium dodecyl sulfate polyacrylamide gel hosphor oresis (SDS-PAGE) and transferred onto poly (vinylidene difluoride) (PVDF) membranes (0.45 μm). The membranes were blocked by bovine albumin and then incubated with antibody over night at 4 ^{o}C. After washing with TBST, the membranes were incubated with horseradish peroxidase-conjugated secondary antibodies for 1 h at room temperature. The proteins on the membranes were visualized by Chemiluminescence Imaging system (ChemiScope 6000 Pro, China). The intensity of immunoreactive bands was quantified by ImageJ software. The primary antibody included hosphor-MEK1/2 (Ser217/221) (1:1000, Cell Signaling, #9154), hosphor-p44/42 MAPK (Erk1/2) (Thr202/Tyr204) (1:1000, Cell Signaling, #4370), MEK1/2 (1:1000, Cell Signaling, # 4694), p44/42 MAPK (Erk1/2) (1:1000, Cell Signaling, # 4695) and β-actin (1:2000, Abcam, ab8226). The secondary antibody included HRP-labeled goat anti-mouse IgG (1:2000, Beyotime, A0216) and HRP-labeled goat anti-rabbit IgG (1:2000, Beyotime, A0208).

Supplementary figures

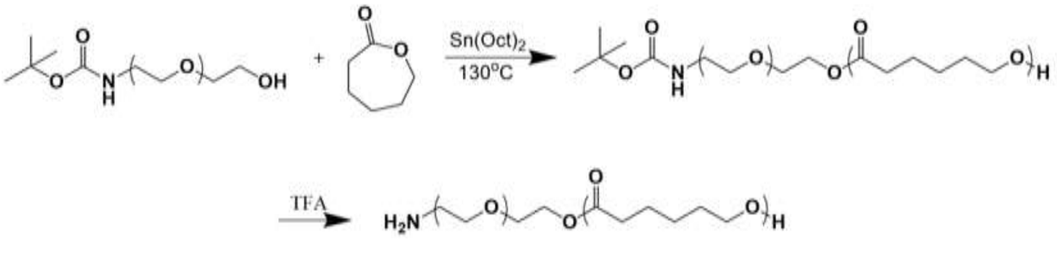

Figure S1. The synthetic process of block copolymer PCN.

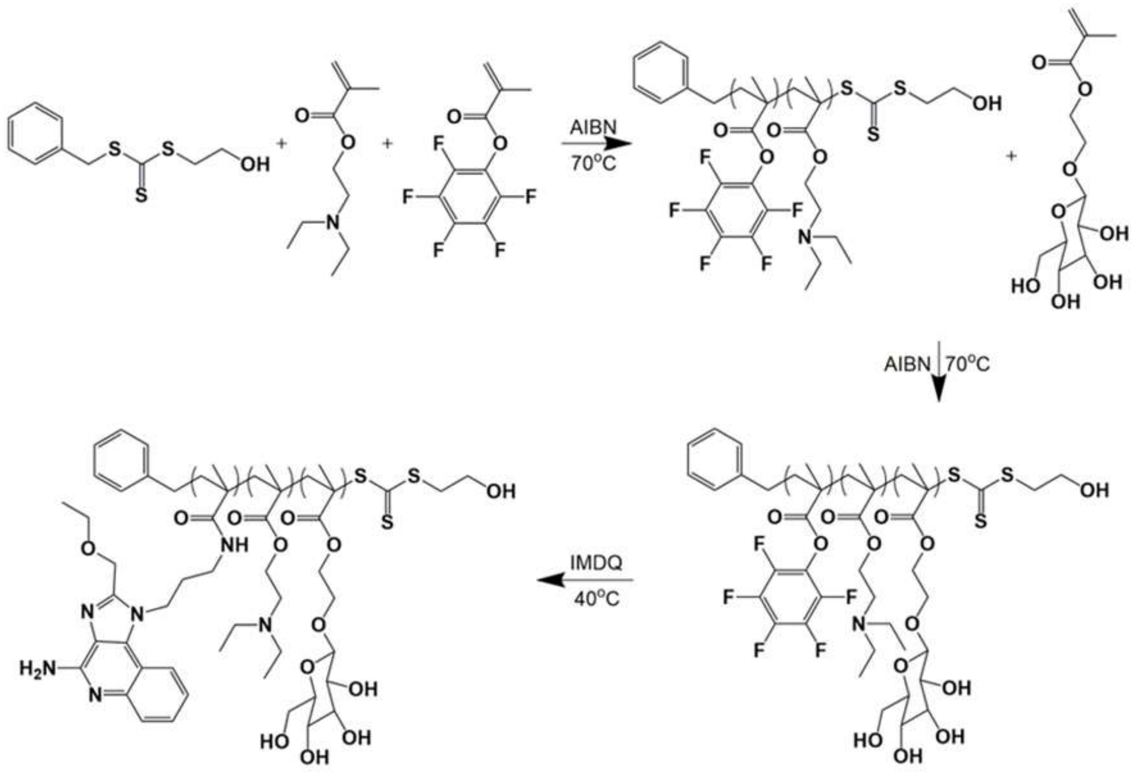

Figure S2. The synthetic process of block copolymer p(Man-IMDQ).

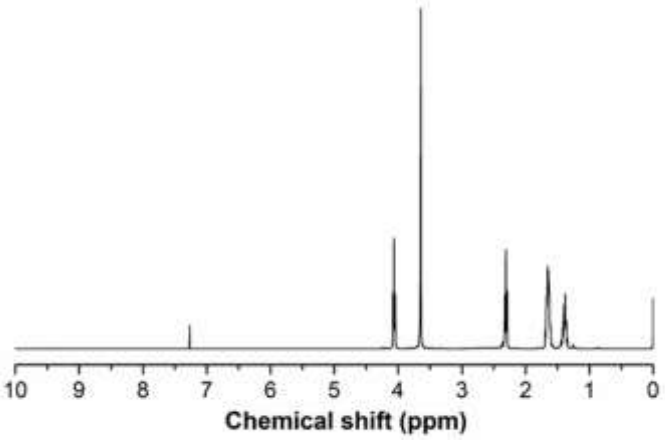

Figure S3. Representative ^{1}H NMR spectrum of PCN.

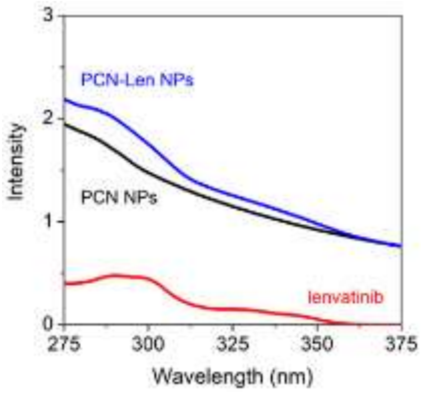

Figure S4. UV-Vis spectroscopy of PCN-Len NPs, PCN NPs and lenvatinib in solution (PCN NPs concentration, 1 mg/mL; lenvatinib concentration, 20 μg/mL).

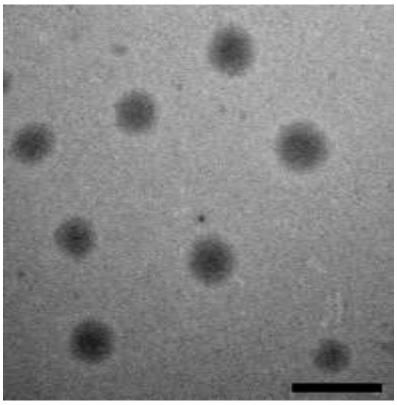

Figure S5. Representative TEM image of PCN-Len NPs. Scale bar, 500 nm.

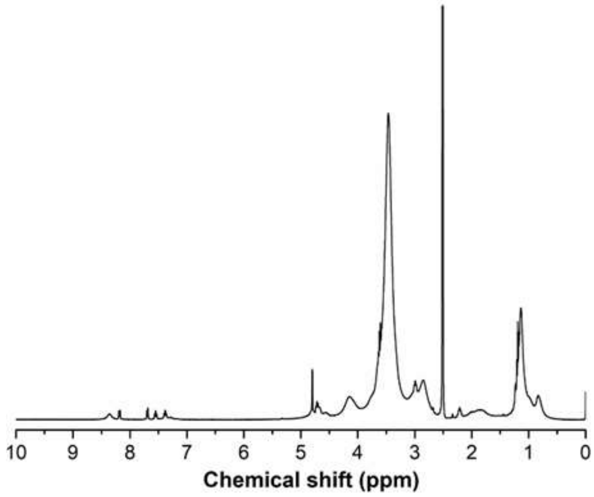

Figure S6. Representative ^{1}H NMR spectrum of p(Man-IMDQ).

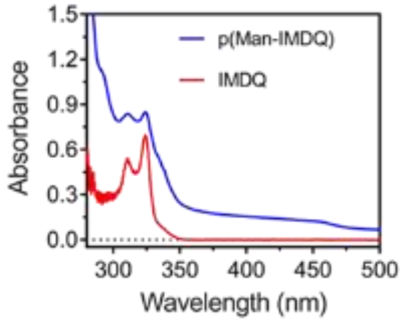

Figure S7. UV-Vis spectroscopy of IMDQ (0.04 mg/ml) and p(Man-IMDQ) in solution.

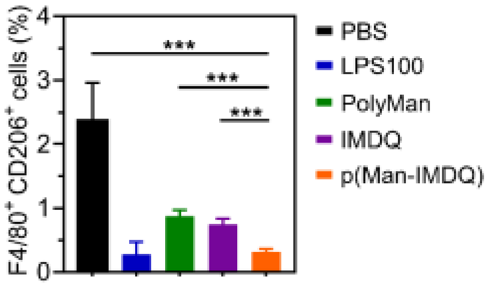

Figure S8. Statistical analysis of the percentage of F4/80$^+$ CD206$^+$ at the BMDMs. Data represent mean ± SD (n = 3).

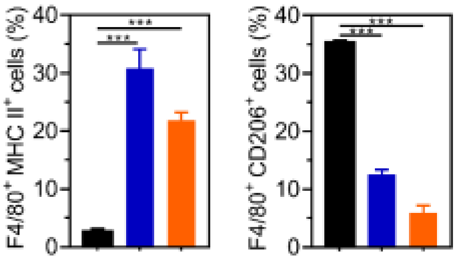

Figure S9. Statistical analysis of the percentage of F4/80$^+$ CD206$^+$ and F4/80$^+$ MHC II$^+$ at the BMDM. Data represent mean ± SD (n = 3).

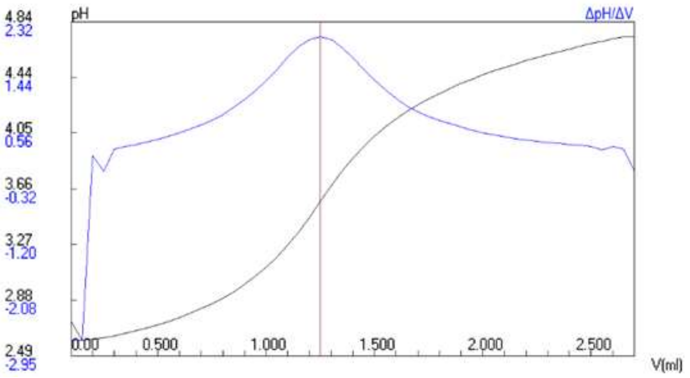

Figure S10. Colorimetric hydroxylamine titration analysis of dextran oxidation. Black line: DX titration with hydroxyl-amine. Red dash line: the first derivative of the titration was

utilized to determine the equivalence point.

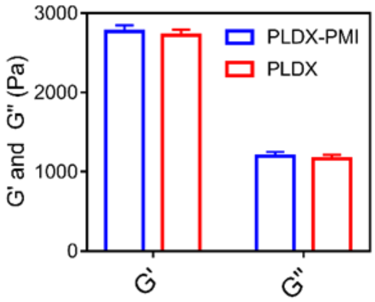

Figure S11. G' and G" values of PLDX-PMI and PLDX by oscillatory shear rheology (shear strain, 1%; angular frequency, 1 rad/s). Data represent mean ± SD (n = 3).

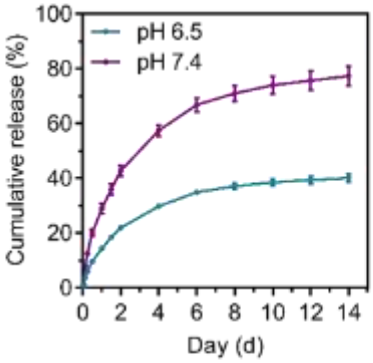

Figure S12. In vitro cumulative release of lenvatinib from the PLDX hydrogel in PBS at pH 7.4 and pH 6.5. Data represent mean ± SD (n=3).

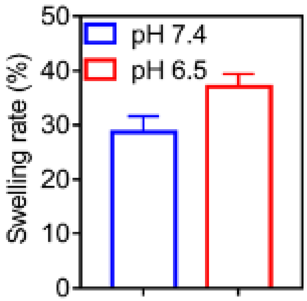

Figure S13. Swelling behavior of PLDX hydrogels in PBS at pH 7.4 and 6.5. Data represent mean ± SD (n = 3).

HUVECs-Fluorescent area/total area		
GROUP	%-AM	%-PI
PDX	15.04	0.01
PCN-Len NPs	1.30	0.64
PLDX-PMI	1.18	0.71

Hepa1-6-Fluorescent area/total area		
GROUP	%-AM	%-PI
PDX	47.34	0.03
PCN-Len NPs	3.82	3.77
PLDX-PMI	7.51	6.17

Figure S14. Quantitative analysis of representative live/dead fluorescent staining of PDX, PCN-Len NPs and PLDX-PMI treated HUVECs or Hepa1-6 cells.

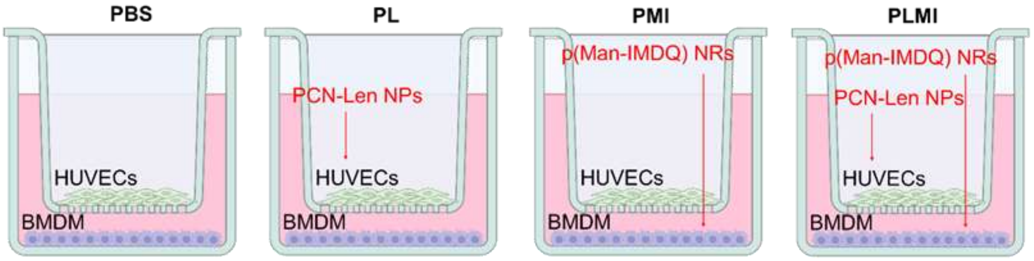

Figure S15. The schematic diagram of BMDM and HUVECs treated with different groups using the transwell system.

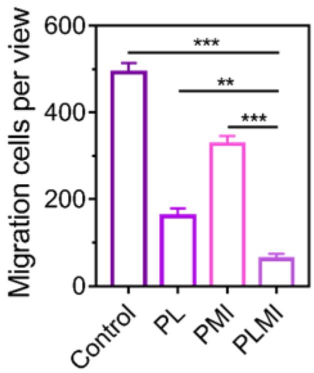

Figure S16. Quantification of inhibition of HUVECs migration treated with different treatments by transwell assay. Data represent mean ± SDs (n=3).

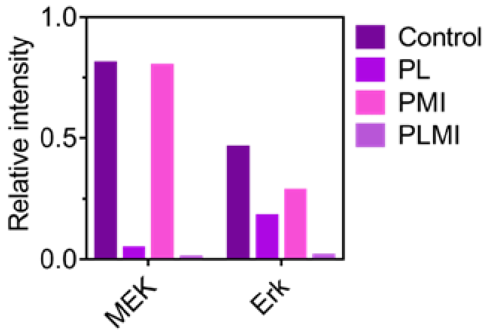

Figure S17. Relative protein expression level of p-MEK and p-Erk in HUVECs analyzed by ImageJ software.

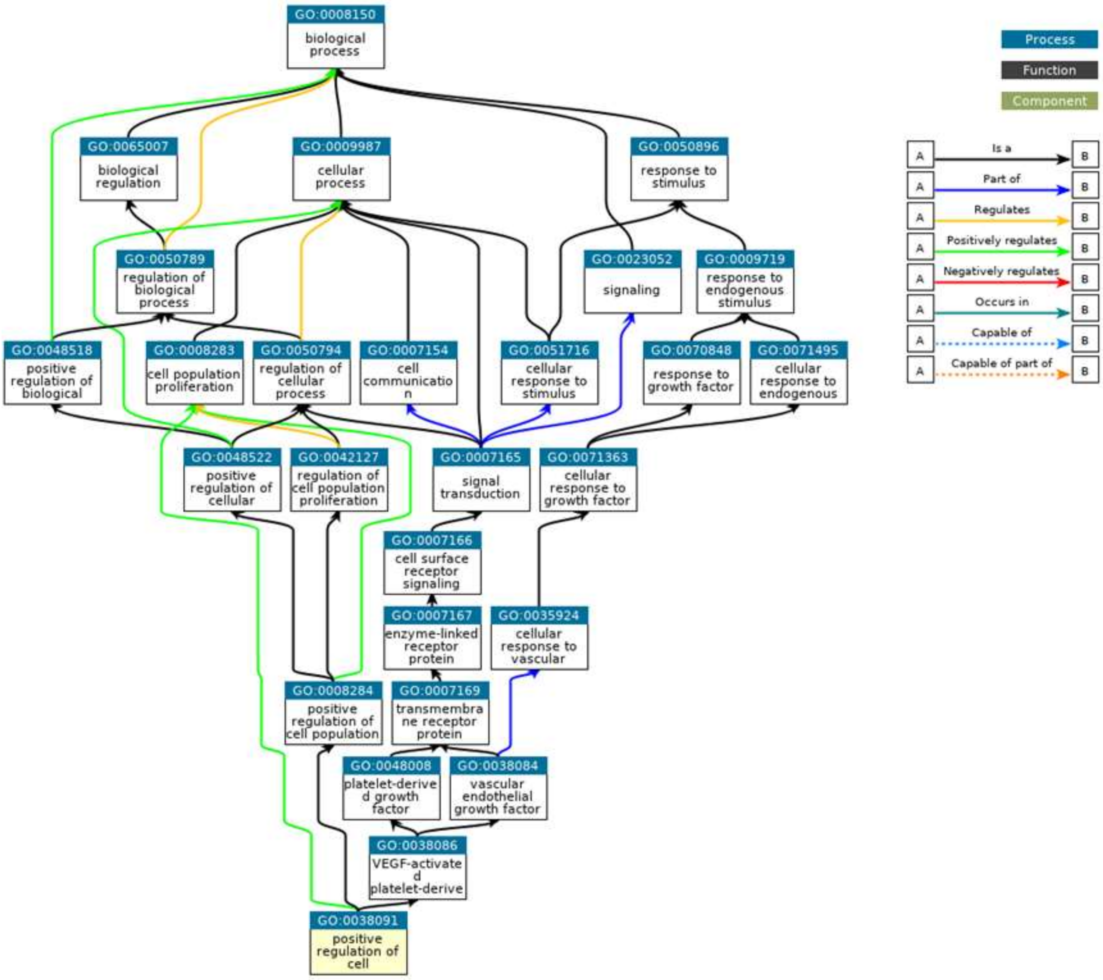

Figure S18. The results of functional enrichment analysis downloaded from DAVID database. The cluster of GO 0048407.

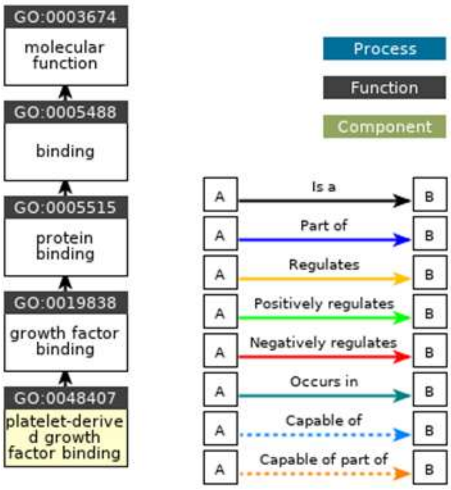

Figure S19. The results of functional enrichment analysis downloaded from DAVID database. The cluster of GO0038091.

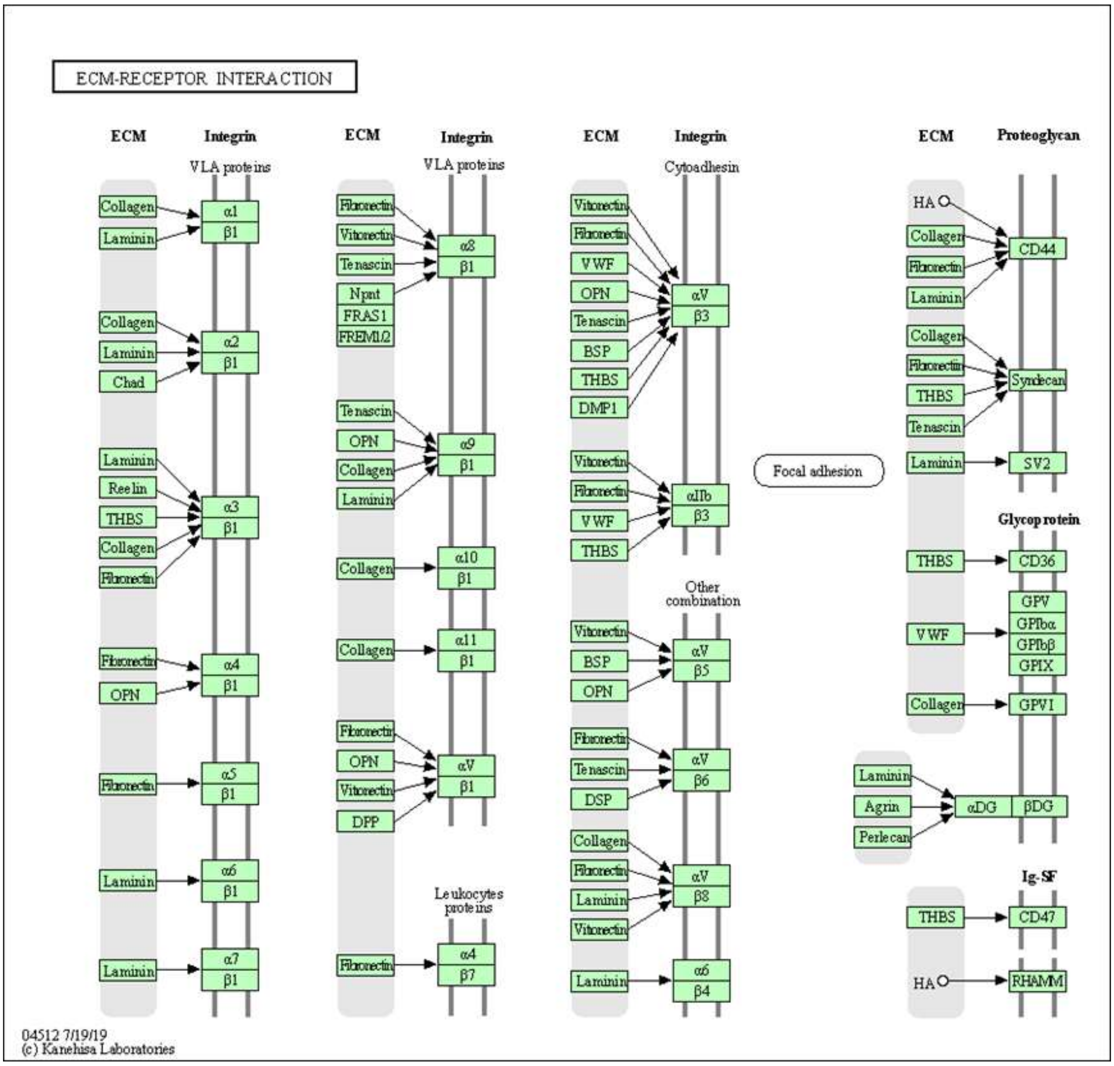

Figure S20. The results of functional enrichment analysis downloaded from DAVID database. The detail of ECM receptor interaction pathway.

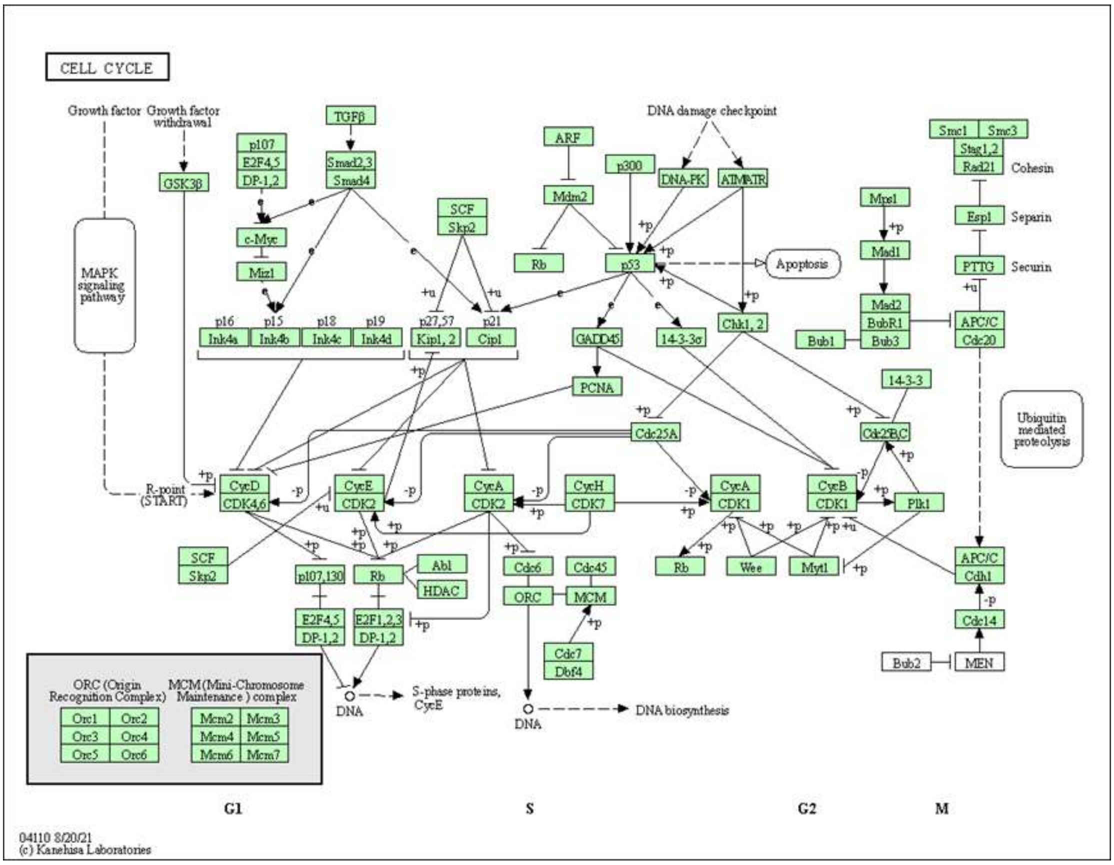

Figure S21. The results of functional enrichment analysis downloaded from DAVID database. The detail of cell cycle pathway.

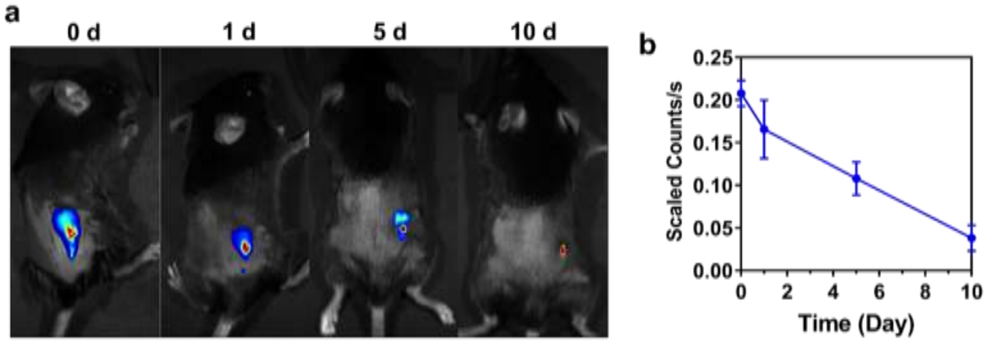

Figure S22. (a) Time-dependent in vivo fluorescence decay of mice after subcutaneous injection with Cy5-labeled PLDX hydrogel. (b) The fluorescence intensity of Cy5-labeled PLDX at different time points after injection. Data represent mean ± SD (n = 3).

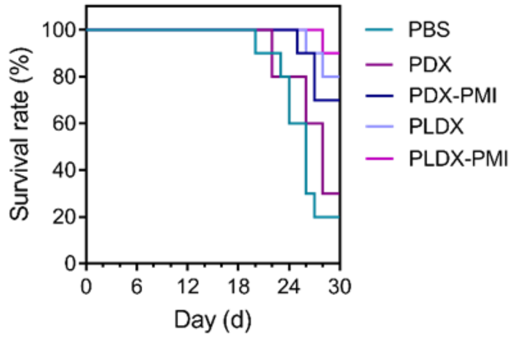

Figure S23. Survival curves of mice bearing orthotopic hepatocellular carcinoma.

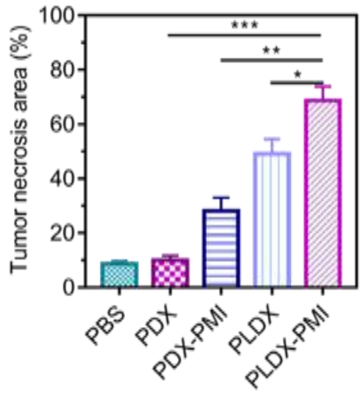

Figure S24. Quantification of the apoptosis area in the tumors. Data represent mean ± SD (n=3).

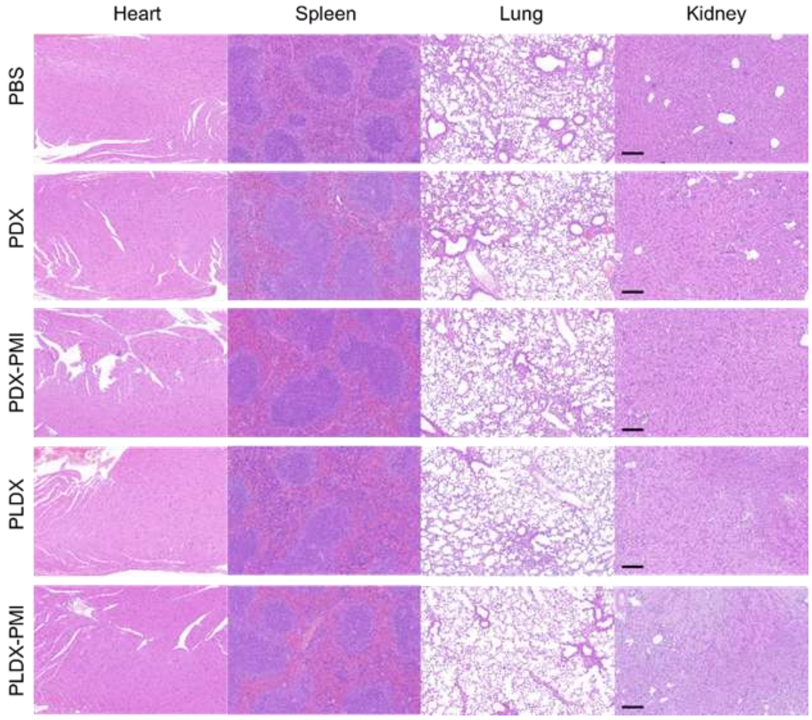

Figure S25. H&E staining of major organs of mice treated with different groups for in vivo

biosafety. Scale bar, 200 μm.

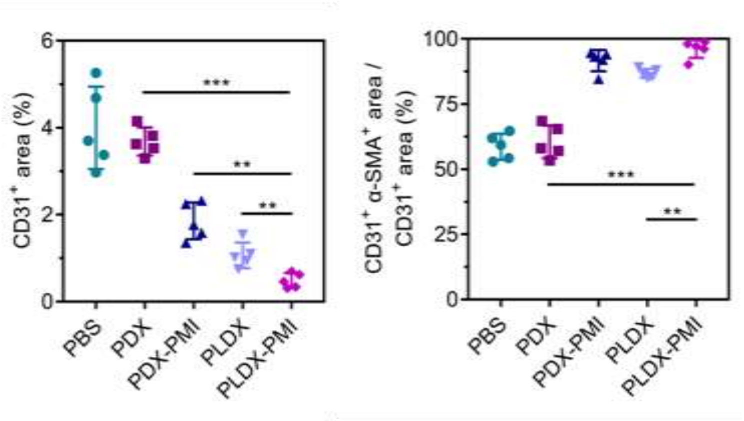

Figure S26. CD31^{+} microvessel areas and CD3^{+} α-SMA^{+}/ CD3^{+} vessel areas in tumors. Data represent mean ± SD (n=5).

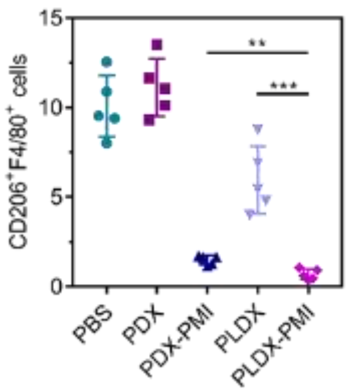

Figure S27. Quantitative analysis of CD206^{+}F4/80^{+} macrophages in tumors. Data represent mean ± SD (n=5).

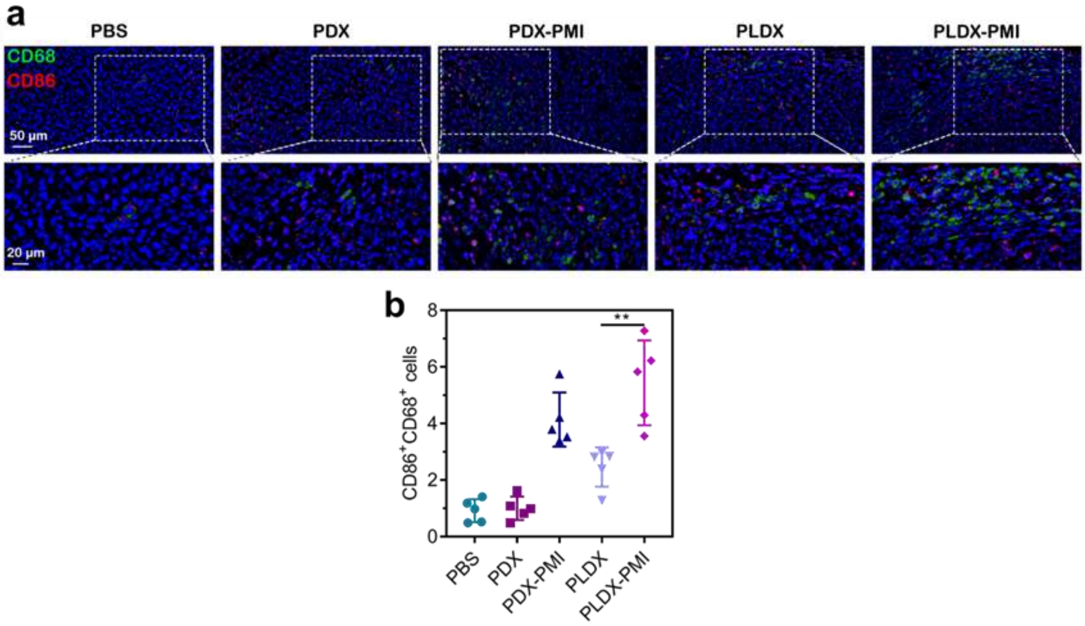

Figure S28. a) Representative images of CD86 (red) / CD68 (green) immunofluorescence staining of tumor tissues. b) Quantitative analysis of $CD86^{+}CD68^{+}$ macrophages in tumors. Data represent mean ± SD (n=5).

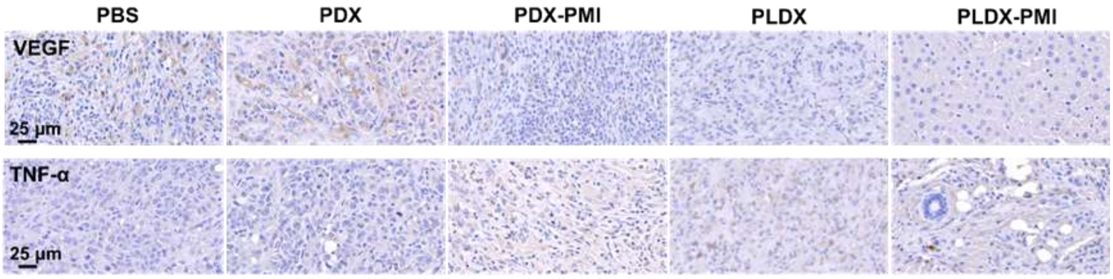

Figure S29. Representative IHC images of VEGF and TNF-α expressions in tumor sections from PBS, PDX, PDX-PMI, PLDX and PLDX-PMI-treated mice.

References

[1] X. Liu, Q. Su, H. Song, X. Shi, Y. Zhang, C. Zhang, P. Huang, A. Dong, D. Kong, W. Wang, *Biomaterials* **2021**, 275, 120921.

[2] L. Du, C. Wang, L. Meng, Q. Cheng, J. Zhou, X. Wang, D. Zhao, J. Zhang, L. Deng, Z. Liang, A. Dong, H. Cao, *Biomaterials* **2018**, 176, 84.
